# Proteomic Analysis of Lipid Droplets from Arabidopsis Aging Leaves Brings New Insight into Their Biogenesis and Functions

**DOI:** 10.3389/fpls.2017.00894

**Published:** 2017-05-29

**Authors:** Lysiane Brocard, Françoise Immel, Denis Coulon, Nicolas Esnay, Karine Tuphile, Stéphanie Pascal, Stéphane Claverol, Laëtitia Fouillen, Jean-Jacques Bessoule, Claire Bréhélin

**Affiliations:** ^1^Plant Imaging Platform, Bordeaux Imaging Center, UMS 3420 Centre National de la Recherche Scientifique, US4 Institut National de la Santé et de la Recherche Médicale, University of BordeauxBordeaux, France; ^2^Laboratory of Membrane Biogenesis, Centre National de la Recherche Scientifique, UMR 5200Villenave d'Ornon, France; ^3^Laboratory of Membrane Biogenesis, University of Bordeaux, UMR 5200Villenave d'Ornon, France; ^4^Bordeaux INPTalence, France; ^5^Proteome Platform, Functional Genomic Center of Bordeaux, University of BordeauxBordeaux, France

**Keywords:** lipid droplet, leaf senescence, label free proteomics, Small rubber particle protein1, electron tomography, ER contact site, ultrastructure, secondary metabolism

## Abstract

Lipid droplets (LDs) are cell compartments specialized for oil storage. Although their role and biogenesis are relatively well documented in seeds, little is known about their composition, structure and function in senescing leaves where they also accumulate. Here, we used a label free quantitative mass spectrometry approach to define the LD proteome of aging Arabidopsis leaves. We found that its composition is highly different from that of seed/cotyledon and identified 28 proteins including 9 enzymes of the secondary metabolism pathways involved in plant defense response. With the exception of the TRIGALACTOSYLDIACYLGLYCEROL2 protein, we did not identify enzymes implicated in lipid metabolism, suggesting that growth of leaf LDs does not occur by local lipid synthesis but rather through contact sites with the endoplasmic reticulum (ER) or other membranes. The two most abundant proteins of the leaf LDs are the CALEOSIN3 and the SMALL RUBBER PARTICLE1 (AtSRP1); both proteins have structural functions and participate in plant response to stress. CALEOSIN3 and AtSRP1 are part of larger protein families, yet no other members were enriched in the LD proteome suggesting a specific role of both proteins in aging leaves. We thus examined the function of AtSRP1 at this developmental stage and found that *AtSRP1* modulates the expression of *CALEOSIN3* in aging leaves. Furthermore, AtSRP1 overexpression induces the accumulation of triacylglycerol with an unusual composition compared to wild-type. We demonstrate that, although *AtSRP1* expression is naturally increased in wild type senescing leaves, its overexpression in senescent transgenic lines induces an over-accumulation of LDs organized in clusters at restricted sites of the ER. Conversely, *atsrp1* knock-down mutants displayed fewer but larger LDs. Together our results reveal that the abundancy of AtSRP1 regulates the neo-formation of LDs during senescence. Using electron tomography, we further provide evidence that LDs in leaves share tenuous physical continuity as well as numerous contact sites with the ER membrane. Thus, our data suggest that leaf LDs are functionally distinct from seed LDs and that their biogenesis is strictly controlled by AtSRP1 at restricted sites of the ER.

## Introduction

In plant cells, neutral lipids constitute a storable source of energy that accumulates in two different compartments: plastoglobules and cytosolic lipid droplets, also known as oil bodies. In contrast to plant-specific plastoglobules, cytosolic lipid droplets (LDs) are described in every organism, from bacteria to human (Yang et al., 2012). LDs are dynamic organelles controlling neutral lipid metabolism and storage. As such, they participate in many critical cellular pathways including lipid transport between organelles (Bartz et al., 2007), protein metabolism (Cermelli et al., 2006; Welte, 2015), or protein degradation (Ohsaki et al., 2006; Welte, 2007; Farese and Walther, 2009; Moldavski et al., 2015). Consequently, dysfunctions in LD biology lead to several metabolic human diseases (Krahmer et al., 2013). In plants, lipid droplets are essential for reproduction by storing oil in seeds, which will be used as source of energy during the post-germinative growth until acquisition of photo-autotrophism. In leaves, LDs accumulate during senescence and transiently store lipids such as steryl esters and triacylglycerol (TAG) derived from the dismantlement of membranes (Chapman et al., 2013; Troncoso-Ponce et al., 2013; Shimada et al., 2015). Yet, the composition, structure and functions of lipid droplets present in vegetative organs, such as leaves or roots (Murphy, 2001; Lersten et al., 2006) remain elusive.

LDs and plastoglobules share a similar structure. They are composed of a central core of neutral lipids, mainly TAGs and lipid-esters (steryl-ester and phytyl ester for LDs and plastoglobules, respectively), covered by a monolayer of polar lipids. While polar lipids found in plastoglobules directly derive from the outer leaflet of the thylakoid membrane, that of LDs derive from the endoplasmic reticulum (ER) membrane (Austin et al., 2006; Jacquier et al., 2011; Kassan et al., 2013; Choudhary et al., 2015). Studies in yeast and animal cells as well as developing seedlings provided current models for LDs biogenesis (reviewed in Chapman et al., 2012; Wilfling et al., 2014). Lipid droplets can arise from previously existing LDs or be formed *de novo*. During *de novo* formation, neutral lipids are synthesized at the ER and subsequently accumulate between the two leaflets of one ER membrane forming lens-like structures that gradually bud from the membrane. LDs will eventually detach from the ER, however it is still unclear whether this last step is systematic or not (Jacquier et al., 2011; Hashemi and Goodman, 2015). In plant leaves, the mechanism for LD biogenesis is not documented. Yet, based on what is found in seeds and other organisms, it is assumed that they derive from the ER.

The characterization of the seed LD proteome has brought crucial information toward the understanding of their structure and functions. To date, less than a dozen proteomic studies have been conducted on purified seed lipid droplets of diverse species (Jolivet et al., 2013; Liu et al., 2015), that identified between 8 and 30 proteins. Oleosins are systematically associated with seed lipid droplets; these proteins are involved in the maintenance of the LD structure, notably by preventing their coalescence during seed maturation. Oleosins are major proteins in LDs of reproductive organs such as seeds and stamens, but are absent from vegetative tissues. For example, in Arabidopsis, 16 oleosin isoforms are present, but none of the encoding genes is expressed in leaves (Kim et al., 2002). Thus, other proteins must play structural functions in vegetative lipid droplets. Caleosins are other LD structural proteins described in seeds as well as in vegetative tissues. Their implication in the maintenance of lipid droplet structure has mainly been demonstrated by *in vitro* assays on artificial lipid droplets (Liu et al., 2009; De Domenico et al., 2011); they are also involved in fatty acid degradation or oxylipin synthesis via their peroxygenase activity (Chapman et al., 2012). Other proteins frequently identified in seed lipid droplets include steroleosins that are involved in sterol and brassinosteroid metabolism. In fact, diverse proteomic approaches of seed LDs identified proteins involved in lipid metabolism, in particular in TAG mobilization during seed germination, as well as in stress response, intracellular transport, and protein biosynthesis (reviewed in Jolivet et al., 2013). In contrast to the seed LDs, the comprehensive protein composition of leaf LDs remains unresolved and only a few proteins have been described to localize at these compartments (Aubert et al., 2010; Shimada and Hara-Nishimura, 2015). Shimada et al. (2014) showed that the caleosin AtCLO3/RD20/PGX3 (At2g33380—thereafter simplified in AtCLO3) interacts with an α-dioxygenase (α-DOX1—At3g01420) in Arabidopsis leaf lipid droplets. This interaction catalyzes the formation of an antifungal phytoalexin, implying a role for leaf lipid droplets in plant defense against fungi. In addition, the TAG lipase Sugar Dependent1 (SDP1), which initiates oil breakdown during germination, localizes to cotyledon lipid droplets during germination (Eastmond, 2006; Thazar-Poulot et al., 2015). However, this localization was described in germinating seedlings, and is therefore associated with seed lipid droplets rather than vegetative lipid droplets from true leaves. During the process of our study, two manuscripts, published concomitantly, demonstrated the localization of a new class of proteins, named either AtSRP for Small Rubber Particle or LDAP for Lipid Droplet Associated Proteins in the LDs of Arabidopsis cotyledon and leaf (Gidda et al., 2016; Kim et al., 2016). These proteins share similarities with the *Hevea brasiliensis* SRPP (Small rubber particle protein) proteins which participate in the maintenance of the structure of rubber particles (Hillebrand et al., 2012), and were previously identified in LDs of the avocado mesocarp (Horn et al., 2013). Gidda et al. (2016) suggested that, during post-germinative growth, the SRP proteins replace oleosins at the LD surface, which are degraded during germination. Yet, while oleosins are highly abundant proteins in seed LDs, the level of SRP proteins in vegetative LDs remains to be determined.

The abundance of leaf LDs dramatically increases during senescence (Shimada et al., 2015). Yet, apart from their role in lipid storage and their implication in the production of an antifungal phytoalexin (Shimada et al., 2014; Shimada and Hara-Nishimura, 2015), little is known about their composition and functions at this developmental stage. Understanding the structure and biogenesis of LDs in senescing leaves will therefore shed new light onto their role in plant aging. In fact, besides SRP, CLO3, and α-DOX1, it is most likely that other proteins associate to lipid droplets, in particular in senescent leaves. We reasoned that a quantitative and qualitative profile of the LD proteome in this tissue would bring new details into the molecular requirements for LD formation and functions. We thus purified vegetative LDs from Arabidopsis aging leaves and performed a label-free quantitative proteomic approach to determine the proteome of these organelles. Proteins were defined as component of the LD core proteome when they were found enriched in the LD fraction compared to any other cell fraction, whatever their abundances within the LD fraction. We found that the protein composition of leaf LDs is quite different from that of seed LDs. In particular, it is strictly deprived of enzymes involved in lipid metabolism. By determining the abundance of each protein within this LD core proteome, we show that the two most abundant proteins in aging leaf lipid droplets are the previously characterized caleosin AtCLO3, and one protein of the small rubber particle protein family, AtSRP1/LDAP1 (At1g67360). In addition, our proteomics data suggest a new role of aging leaf lipid droplets in several pathways for secondary metabolite biosynthesis and plant resistance to stress, notably herbivory and pathogen resistances. Using laser scanning confocal and electron microscopy, we demonstrate that AtSRP1 is sufficient to induce lipid droplet biogenesis in leaf at restricted sites of the ER, and necessary to regulate LD size and number. We took advantage of this property to establish electron tomography of cryofixed leaf LDs and demonstrate that they share numerous contact sites as well as some continuity with the ER membrane.

## Materials and methods

### Plant material and culture conditions

For LD preparation, *Arabidopsis thaliana* ecotype Columbia (Col-0) were grown at high density on soil in a phytotron in long day conditions (16 h light/8 h dark), 100 μmol of photon.m^−2^.s^−1^ at 20–22°C and 65% humidity. For nitrogen starvation assays, 10-day-old plants grown on half strength Murashige and Skoog (MS) medium were transplanted on synthetic minimal medium deprived of nitrogen [0.8% Agarose, 1% sucrose, 1 mM MgSO_4_, 1 mM KH_2_PO_4_, 25 μM Fe-EDTA, 35 μM H_3_BO_3_, 7 μM MnCl_2_, 0.25 μM CuSO_4_, 0.5 μM ZnSO_4_, 0.1 μM Na_2_MoO_4_, 5 μM NaCl, 5 nM CoCl_2_, 1 mM CaCl_2_, and 2.5 mM KCl] (Gaude et al., 2007) and grown for 6–7 additional days. For lipid analysis, seeds were first germinated on half strength MS medium, and then transplanted in individual pots in growth chamber in long day conditions, at 20–22°C and 40% humidity. The *atsrp1_1* (SALK_148081) and *atsrp1_2* (GK-309G05) T-DNA mutants were obtained from the ABRC seed stock center, and homozygous lines were selected by PCR genotyping (primer sequences are provided in Table S1). For transient transformation, *Nicotiana benthamiana* leaves or 4-day-old *A. thaliana* seedlings were infiltrated with *Agrobacterium tumefaciens* cell cultures as described in Perraki et al. (2014) or Marais et al. (2015) respectively.

### Cloning and transgenic plants

ORFs clones of *AtSRP1* (U17438) and *AtULP* (U15698) in *pENTR* vector were obtained from ABRC clone center and verified by sequencing. *AtULP* clone contained a non-silent mutation (L_63_ → M_63_) that was corrected by PCR quick-change site directed mutagenesis. ORFs with or without the stop codon were subsequently transferred by GATEWAY® recombinational cloning technology in diverse destination vectors under the 35S promoter in pGWB661 and pGWB660 (Nakamura et al., 2010), and under the *UBQ10* promoter in *pUBNYFP* or *pUBCYFP* (Grefen et al., 2010) to obtain *TagRFP-AtSRP1, AtSRP1-TagRFP, YFP-AtSRP1*, and *AtSRP1-YFP* constructs respectively. The resulting plasmids were introduced into Arabidopsis plants by an Agrobacterium transfection method (Clough and Bent, 1998). Two or three confirmed lines, numbered #a to c, were used for the different experiments. Sequences of primers used for cloning are given in Table S1.

### Preparation of lipid droplet fractions

For lipid droplet purification, *c.a*. 150 g of 6-week-old *A. thaliana* leaves were homogenized in cold G-20 buffer (100 mM Tricine (pH7.5), 10 mM KCl, 1 mM EDTA, 1 mM PMSF, plant protease inhibitor cocktail (Sigma), and 20% sucrose) using a Waring blender. The homogenized plant material was filtered through two layers of cheese cloth and one layer of Miracloth (Calbiochem) and split into three equal portions that were processed in parallel to obtain three technical replicates. A scheme representing the purification process is provided in Figure S1. Each portion of the filtrate was centrifuged for 30 min at 10,000 rpm. The floating lipid pad was kept, and the soluble fraction centrifuged at 100,000 g for 30 min. The floating lipid pad was collected and pooled with the previous one. The pellet and the soluble fraction above the floating lipid pad were used for isolation of membrane and soluble proteins respectively. The pooled floating lipid pads were resuspended in 10 mL G-20 buffer using a Potter homogenizer, distributed in 2 centrifuge tubes, covered with 2 mL of G-15 buffer (G-buffer with 15% sucrose) and 5 mL of G-5 buffer (G buffer with 5% sucrose), and centrifuged 50 min at 150,000 g. The two floating lipid pads were pooled, resuspended in 5 mL G-20 buffer, and centrifuged similarly with G-15 and G-5 buffers. Finally, each floating lipid pad was resuspended in 5 mL G-20 buffer with a Potter homogenizer, covered with 7 mL G-0 buffer (without sucrose), and centrifuged 50 min at 150,000 g. The three resulting floating lipid pads from the three starting portions were stored at −80°C. Plastoglobules were purified according to the protocol described by Besagni et al. (2011).

### Protein analysis

Proteins were extracted according to Rensink et al. (1998) and concentrated by chloroforme-methanol precipitation (Wessel and Flugge, 1984). For immunoblot experiments, proteins were separated by SDS-PAGE (Sodium Dodecyl Sulfate—PolyAcrylamide Gel Electrophoresis) and blotted onto nitrocellulose membrane for immunodetection. Blots were probed with an antibody raised against GFP (Roche), or sera raised against plastoglobule, ER, Golgi or thylakoid markers, respectively AtFBN1b (Vidi et al., 2006), BiP (Höfte and Chrispeels, 1992), AtMemb11 (Marais et al., 2015), or P16 (Vallon et al., 1986). Proteins were quantified either according to the Bradford method (Bradford, 1976) or the instructions of the QuantiPro BCA assay kit manual (Sigma).

### Label-free quantitative data analysis

Proteins were identified by mass spectrometry and relatively quantified by a label-free approach (for detailed description see Appendix S1). Ten microgram of each fraction were loaded onto a 10% acrylamide SDS-gel. Trypsin digested peptide mixture was analyzed on a Ultimate 3000 nanoLC system (Dionex, Amsterdam, The Netherlands) coupled to a LTQ-Orbitrap XL mass spectrometer (Thermo Fisher Scientific, San Jose, USA). Data were searched by SEQUEST through Proteome Discoverer 1.4 (Thermo Fisher Scientific, San Jose, USA) against the TAIR (The Arabidopsis Information Resource) protein database. Raw LC-MS/MS data were imported in Progenesis QI 2.0 (Nonlinear Dynamics Ltd, Newcastle, UK). Protein abundance was calculated according to two methods: when comparing protein abundance between samples the protein abundance was calculated based on peptide uniqueness, as preconized by the Progenesis tool, while the TOP3 method (sum of the three most abundant peptides), which allows an absolute quantification of the protein (Silva et al., 2006), was chosen to compare abundance of the different proteins in a given sample. If the same set of peptides could be used to identify several different proteins, these proteins were grouped and counted as one protein for quantification. The relative normalized abundance of each protein in a single sample was calculated from its normalized abundance relatively to the sum normalized abundance of all the peptides in the sample. Average relative normalized abundances were calculated based on the triplicates of the enrichment ratios between LD and each of the other fractions calculated for each protein. Quantitative data were considered for proteins quantified by a minimum of 2 peptides. The mass spectrometry proteomics data have been deposited to the ProteomeXchange Consortium via the PRIDE (Vizcaíno et al., 2016) partner repository with the dataset identifier PXD006113.

### Lipid analysis

Lipids were extracted and purified according to Folch et al. (1957). The lipids were then resuspended in chloroform:methanol (2:1, v/v) solution and applied onto a silica-coated chromatography plate (Merck) with lipid standards. Lipids were separated with a single (leaf lipid composition) or double (purified LD analysis) thin layer chromatography (TLC): polar lipids were first separated with a Vitiello-Zanetta solvent mixture (Vitiello and Zanetta, 1978). The neutral lipids were then separated according to Juguelin et al. (1986). The lipids were visualized with primuline under UV light. After fatty acid trans-esterification, lipids were quantified by gas chromatography (Agilent 7890 gas chromatograph equipped with a Carbowax column, Alltech Associates, Deerfield, IL, USA) coupled to a flame ionization detector (GC-FID).

### Confocal microscopy and BODIPY^493/503^ coloration

Observations were performed on a confocal microscope Leica TCS SP2. When needed, leaf tissues were stained with a neutral lipid-specific fluorescent dye, BODIPY^493/503^ (MolecularProbes), at a final concentration of 1 μg/mL for 5 min in the dark. The acquisitions with objective X63 were taken sequentially on each channel with the following acquisition settings: excitation wavelength of 514 nm and emission of 520–550 nm band pass for YFP, excitation wavelength of 543 nm and emission of 600–650 nm band pass for TagRFP, excitation wavelength of 514 nm and emission filter of 520–540 nm band pass for BODIPY^493/503^, excitation wavelength of 514 nm and emission of 655–690 nm or 695–750 nm band pass for chlorophyll autofluorescence. Stack image deconvolution was achieved with Autoquant software with blind PSF mode and five iterations.

### TEM imagery and tomography

Leaves were chemically fixed by 2.5% glutaraldehyde in phosphate Buffer 0.1 M pH 7.2 overnight at 4°C and post-fixed by 1% OsO_4_ for 2 h at room temperature. After dehydration, inclusion in SPURR (Electron Microscopy Science) resin was progressively performed. To finish, the samples were polymerized at 70°C for 16 h. Cryofixation was performed on the first leaves of 6-day-old plants. The samples were high pressure frozen (HPF) with an EM-PACT1 (Leica) device and 20% BSA in half MS medium was used as cryoprotectant. Freeze-substitution steps were achieved in an AFS2 (Leica) system. Samples were first incubated at −90°C into a glutaraldehyde 0.5%, osmium tetroxide 2%, uranyl acetate 0.1% in pure acetone for 48 h. For IEM (Immuno electron microscopy), osmium tetroxyde and glutaraldehyde were omitted. Then a progressive raise of the temperature of 3°C/h was initiated until −50°C is reached, and samples were progressively incubated in HM20 Lowicryl resin (Electron Microscopy Science) and polymerized under UV for 48 h at −50°C followed by 48 h at 20°C. Ultrathin sections of 70–80 nm and 180 nm thickness were made with ultramicrotome Leica EM UC7. IEM was made according to Grison et al. (2015). GFP antibodies (Tp401, Torrey Pines Biolabs) were diluted 1/500 before immuno-gold-labeling with anti-rabbit conjugated with 5 nm colloidal gold (Tebu) diluted 1/30. The transmission electron microscope FEI Tecnai G2 Spirit TWIN 120 kV equipped with a CCD 16 Mpixels Eagle 4k camera was used for acquisition (120 kV). ImageJ software (Schneider et al., 2012) was used for image analysis. For electron tomography, FEI software was used for tilt series acquisition. Alignement and backprojection steps were respectively achieved with IMOD and tomoJ softwares. OSART algorithm with 100 iterations was used.

### RNA extraction and quantitative RT-PCR

RNAs were extracted with the RNeasy Plant Mini kit (Qiagen) on green parts of plants, treated by DNAse (Turbo DNA-free™ kit, Ambion) and quantified with the NanoDrop 2000 system (Thermo Scientific). Reverse transcriptase reaction was carried out on 143 ng of extracted RNA with SSII Reverse Transcriptase (Invitrogen). For quantitative PCR, three reference expression genes were used: *actin* (At1g49240), *GAPDH* (At1g13440) and *eIF4A-1* (At3g13920). The sequence of the primers is provided in Table S1. Three wells per condition were quantified and efficiency test was performed on primers. Transcript abundance in samples was determined using a comparative cycle threshold (Ct) method on the CFX96 Touch™ Real-Time PCR Detection System (Bio-Rad). The relative abundance of the three genes of reference in each sample was determined and used to normalize for differences of total RNA level according to the method described by Vandesompele et al. (2002).

## Results and discussion

### Purification of vegetative LDs from arabidopsis aging leaves

**Figure 1:**
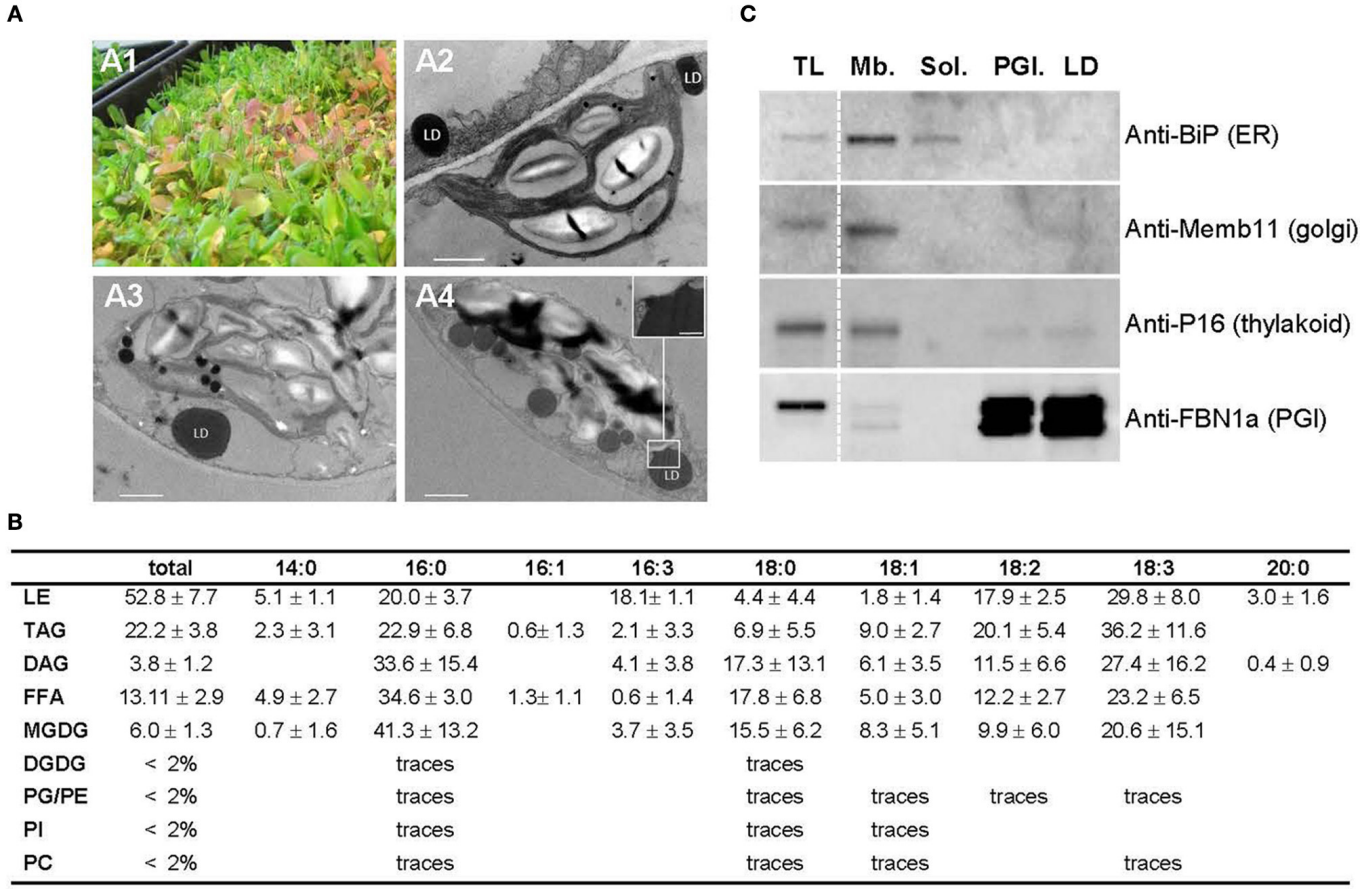
Preparation of aging leaf lipid droplet fraction. **(A)** Six week old Arabidopsis leaves accumulate lipid droplets. **(A1)** 6 week old leaves grown under long day conditions; **(A2–A4)**: details of the ultrastructure observed by TEM of a parenchyma cell from green **(A2)**, purple **(A3)**, and red **(A4)** leaves showing lipid droplet in close proximity to chloroplast. Insert in **(A4)** shows the plastid—LD contact site. Scale bar: 1 μm, except in insert: 200 nm. LD, lipid droplet. **(B)** Lipid composition of Arabidopsis leaf lipid droplets (in % of total fatty acids) *n* = 5. Lipids were extracted from LD fraction, separated by thin layer chromatography, and quantified by GC-FID after transesterification. LE, lipid ester; TAG, triacylglycerol; DAG, Diacylglycerol; FFA, free fatty acid; MGDG, monogalactosyldiacylglycerol; DGDG, digalactosydiacylglycerol; PG, phosphatidylglycerol; PE, phosphatidylethanolamine; PI, phosphatidylinositol; PC, phosphatidylcholine. **(C)** Immunoblot analysis of cell fractions using marker antibodies. Proteins for total leaf (TL), membranous (Mb.), soluble (Sol.), plastoglobules (PGl.), and lipid droplet (LD) fractions were precipitated and 10 μg from each fraction were loaded on SDS-PAGE. After transfer to nitrocellulose, proteins were probed with sera raised against Binding immunoglobulin protein (BiP) as ER marker, Membrin 11 (Memb11) as Golgi marker, P16 as thylakoid marker, and Fibrillin 1a (FBN1a) as plastoglobule marker. Two lines of the blots were cropped (dashed line) for clarity reason, but brightness and contrast balance were applied to every pixels of the whole blots before cropping.

LDs have been described to accumulate in senescing leaves (Parker and Murphy, 1981; Shimada et al., 2015), therefore we chose to harvest aging leaves, from 6 to 7 week old Arabidopsis plants as starting material for the isolation of leaf LDs. Arabidopsis of this age, grown at high density, display a mix of green, purple, red and yellow leaves reminiscent of a stress and senescent phenotype (Figure 1A1). The accumulation of LDs was tested by transmission electron microscopy on chemically fixed leaves (Figures 1A2–A4). Parenchymal cells of aging leaves accumulated, per cell section, 1–7 lipid droplets from 400 to 1,600 nm diameter (with an average of *c.a*. 820 nm) while parenchyma plants of 3 week old green leaves exhibited very little to no LD. In addition to cytosolic LDs, we observed an accumulation of large plastoglobules (*ca*. 310 nm) in plastids, which was more pronounced in red leaves. This is consistent with previous studies showing that plastoglobules are also known to accumulate in response to a variety of stress as well as during senescence (Nacir and Bréhélin, 2013).

LDs were purified by successive centrifugations on sucrose gradients (see Figure S1). Because no antibody specific to leaf LDs was available to test the quality of the resulting fraction by immunoblotting, we determined by TLC GC-FID the lipid composition of the final fraction to confirm the presence of lipid droplets. The LD fraction contained, as expected, mainly neutral lipids with 53% of total fatty acids (FAs) in lipid esters, 22% of FAs in TAG and 13% in free fatty acids (FFAs), and less than 5% of FAs in total phospholipids (Figure 1B). We also detected minor quantities of MGDGs in our LD fraction. MGDGs are lipids typically associated with plastid membranes; yet their composition in LDs differed from that of chloroplast MGDGs, in particular in respect to C16:3 (3.7% in LDs compared to 35% in plastid MGDG, Ajjawi et al., 2011), which is an acyl moieties commonly considered as exclusively plastidial (Li-Beisson et al., 2013). Therefore, only 10% of the MGDG present in LD could be explained by a chloroplast contamination, if any, and a major contamination of the LD sample by plastid membranes can be excluded. In addition, the proportion of phospholipids to neutral lipids in our purified fraction was similar to that of seed LDs (Huang, 1992), demonstrating that it was mainly constituted by lipid droplets and contained, if any, only traces of other organelles. To further test the purity of the LD fraction, we performed immunoblot analyses using markers of the most probable contaminants: Golgi vesicles that may float similarly to the LDs, ER membranes that could stay attached to LDs, and thylakoids, the most abundant membranes in leaves. As expected, the LD fraction did not show any signal with Golgi and ER markers (Figure 1C). A faint signal was observed with the thylakoid marker, suggesting a weak contamination by thylakoid membranes. However, the lipid analysis demonstrated that possible contaminating membranes are far less abundant than neutral lipids in the LD fraction. Furthermore, lipid esters in the LD fraction were constituted of 18.1% C16:3 in addition to 20.0% of C16:0, 17.9% C18:2, and 29.8% C18:3 (Figure 1B), while Arabidopsis leaf steryl esters do not contain any C16:3 (Wewer et al., 2011). On the contrary, fatty acid phytyl esters (FAPEs), described to accumulate in plastoglobules are constituted by 50% of C16:3 and neither C18:2 nor C18:3 (Gaude et al., 2007). This suggests a contamination of the LD fraction by FAPEs originating from plastoglobules. We therefore performed immunoblot assays with a serum raised against the fibrillin/plastoglobulin AtFBN1a/AtPGL35 (At4g04020), a plastoglobule marker (Vidi et al., 2007). The LD fraction showed a signal equivalent to the one obtained with purified plastoglobules. It confirmed that our LD fraction contains plastoglobules in addition to cytosolic lipid droplets. This can be easily explained since plastoglobules have a density similar to LDs, and are also purified by floatation on a sucrose gradient (Besagni et al., 2011). During the purification process chloroplasts were inevitably broken, releasing plastoglobules that were then co-purified with LDs. The plastoglobule proteome has already been well described (Vidi et al., 2006; Ytterberg et al., 2006; Lundquist et al., 2012), with a core proteome of 34 proteins (Nacir and Bréhélin, 2013) that can manually be removed from the LD core proteome, therefore we choose to performed the proteomic analysis on this fraction enriched in LDs and plastoglobules, and deprived of any other major contaminants.

### Defining the leaf LD proteome

In order to establish precisely the leaf LD proteome, we quantified protein abundance by label free quantitative proteomics, and determined the enrichment ratio of each protein in the LD fraction compared to other cell fractions. This should allow eliminating any protein unspecifically detected in LDs (for example contaminant proteins highly abundant in other cell fractions) and identifying with certainty proteins specific to the LD fraction. Arabidopsis leaf LDs were purified from three independent 6 week old cultures. In addition, fractions enriched with soluble or membrane proteins were also prepared from the same starting material. From each independent culture, three technical replicates were analyzed, coming from three gradients performed in parallel (for LD, soluble and membrane fractions) or three different leaves (for total leaf extracts). Proteins within each fraction were thereafter identified and quantified by label free quantitative proteomics. The data obtained are provided in Table S2. Some proteins with a same ontology were grouped under a unique identifier when they share identical peptide sets. To be able to compare the abundance of a protein between the different fractions, we followed a protocol similar to the one previously described by Lundquist et al. (2012) for the identification of the plastoglobule core proteome. First, the relative mass contribution (relative normalized abundance) of each protein within a fraction was calculated from its normalized abundance quantified by the label free method relatively to the sum normalized abundance of all the peptides in the fraction. The averages of relative normalized abundances from triplicates of the same experiment were determined. Second, the enrichment factor between LD and each of the other fractions was obtained for each protein by calculating the ratios of the average of relative normalized abundance in LD to other fractions (see Figures S2, S3 for graphical explanation). Next, these ratios were analyzed according to several criteria to establish a list of proteins enriched in the LD fraction: (1) the LD/total leaf enrichment ratio had to be superior to 6; this threshold was chosen deliberately low (given that the total protein amount in an entire cell is much more than 6 times more abundant than the LD total protein amount of a cell), in order to minimize the risk to exclude major LD proteins that could partition between LDs and other compartments, but sufficient to remove all abundant proteins such as subunits of the photosystem complexes. (2) Proteins with a ratio of LD to soluble proteins and LD to membrane proteins inferior to 1.5 were discarded. This enrichment ratio was chosen as low as possible in order to obtain the most possible exhaustive list of proteins. The resulting data identified plastoglobule proteins in the LD fraction as suspected from our immunoblot analyses, the most abundant being the fibrillins AtFBN4 (At3g23400) and AtFBN1b (At4g04020). Purified plastoglobules highly impoverished in LDs can easily be obtained because plastoglobules are isolated from purified chloroplasts. In order to clearly exclude contaminating plastoglobule proteins from our LD proteome, we purified plastoglobules from the material used for LD purification in one of the three replicates, and the LD/plastoglobule enrichment ratio was determined for each protein. Thirty two of the thirty four plastoglobule proteins defined in Nacir and Bréhélin (2013) were identified both in the plastoglobule and LD fractions (Table S3). In this assay, the known plastoglobule protein with the highest LD/plastoglobule ratio was the kinase ABC1K3 with a ratio of 3.99. Thus proteins with LD/plastoglobule enrichment ratio less than 4 were considered as non-LD proteins but rather plastoglobule ones, and were thus discarded.

Thirty three proteins or groups of proteins matching all the aforementioned criteria in at least two of the three independent experiments were considered enriched in LDs (Table 1). We further excluded five proteins with a previously characterized plastidial localization and/or function (Table S4). Among the 28 proteins assigned to the core LD proteome (Table 1), three were already known to associate with leaf LDs: Aubert et al. (2010) described the co-localization of the caleosin AtCLO3 with lipid droplets in onion epidermal cells and the accumulation of the corresponding transcripts in leaves, especially in response to drought stress. We also identified the peroxidase α-DOX1 that interacts with AtCLO3 in leaf LDs (Shimada et al., 2014), and AtSRP1, one of the three members of the AtSRP/LDAP family (Gidda et al., 2016; Kim et al., 2016) is also present in our proteome. The identification of these already known LD proteins in the core proteome validated our approach. Twenty three other proteins were classified into seven biological processes according to the MapMan tool provided by the Bio-Analytic Resource for Plant Biology (http://bar.utoronto.ca/), UniProt Gene Ontology (Consortium, 2015) and KEGG BRITE database (Kanehisa et al., 2016): cytoskeleton proteins (3), transport (6), stress response (3), biosynthesis of secondary metabolites (6), biosynthesis of amino acids (2), cell wall modification (1), and peptidases (2), while two proteins remained unclassified: an unknown protein (At5g16550) and a HAD superfamily acid phosphatase.

**Table 1:** Aging leaf LD core proteome determined by label free quantitative comparative proteomics.

**Protein name^b^**	**Gene ID^c^**	**Most probable location according to literature and databases^d^**	**Enrichment ratios**									
			**Exp. 1**			**Exp. 2**			**Exp. 3**			
			**LD/S**	**LD/Mb**	**LD/TL (>6)**	**LD/S**	**LD/Mb**	**LD/TL (>6)**	**LD/S**	**LD/Mb**	**LD/TL (>6)**	**LD/PG (>4)**

**KNOWN LIPID DROPLET PROTEINS**^a^												
Caleosin-related family protein (CLO3/RD20)	AT2G33380	LD^(1)^	208.0	30.5	202.7	61.7	21.6	53.7	107.1	36.7	102.1	64.0
Peroxidase superfamily protein (α-DOX1)	AT3G01420	LD^(2)^	2.8	40.2	**3.1**	19.8	21.5	21.7	3.2	3.3	6.6	4.4
Rubber elongation factor protein (SRPP1/LDAP1)	AT1G67360	LD^(3.4)^	114.3	67.1	80.1	26.4	22.0	34.3	66.0	57.8	69.6	83.9
**BIOSYNTHESIS OF SECONDARY METABOLITES**												
Strictosidine synthase 2 (STR2)	AT1G74010	Cytosol/vacuole^(5.6)^	126.4	14.8	79.2	45.7	5.8	22.4	86.3	4.3	19.8	18.4
	AT1G74020											
Farnesylcysteine lyase (FCLY)	AT5G63910	Membranes^(7)^	20.9	7.6	11.9	11.3	9.6	18.3	98.8	3.8	14.9	53.0
2-Oxoglutarate (2OG) and Fe(II)-dependent oxygenase superfamily protein (GSL-OH)	AT2G25450	Cytosol?^(8)^	43.2	8.7	51.1	83.7	4.0	54.0	nd	nd	nd	nd
Nitrile specifier protein 5 (NSP5)	AT5G48180	Cytosol?^(9)^	3.4	125.6	42.2	2.3	2.0	13.3	nd	nd	nd	nd
Cytochrome P450, family 87, subfamily A	AT3G03470	ER^(10)^	9.0	7.4	28.7	15.5	2.6	7.5	nd	nd	nd	nd
Chlorophyllase 1	AT1G19670	Cytosol^(11)^	2.2	6.9	16.8	3.8	11.5	31.6	247.8	**1.2**	7.9	65.9
**BIOSYNTHESIS OF AMINO ACIDS**												
Methionine adenosyltransferase 3 (SAMS3)	AT2G36880	Cytosol^(12)^	9.2	3.6	11.1	21.6	5.3	9.9	4.9	2.8	6.9	30.8
3-Deoxy-d-arabino-heptulosonate 7-phosphate synthase (DHAP)	AT1G22410	Plastid/cytosol^(13–15)^	162.5	51.2	15.3	24.2	8.1	16.2	17.4	6.9	**3.9**	50.1
	AT4G33510											
**STRESS RESPONSE**												
Early-responsive to dehydration 7 (ERD7)	AT2G17840	Unclear	10.2	13.8	10.2	12.3	5.7	8.6	3.7	**1.1**	**0.7**	15.4
Peroxidase superfamily protein	AT5G64110	Unclear	67.7	17.0	176.9	nd	nd	nd	53.7	6.8	41.1	15.4
EXORDIUM like 4	AT5G09440	Unclear	nd	nd	nd	4.0	20.9	10.6	4.7	7.2	10.5	30.2
**CELL WALL MODIFICATION**												
Xyloglucan endotransglucosylase/hydrolase 4	AT2G06850	Cytosol/Extracellular (GFP database)^(16)^	nd	nd	nd	2.4	6.8	10.4	6.1	14.4	8.1	33.2
Probable xyloglucan endotransglucosylase/hydrolase protein 16	AT3G23730											
Xyloglucan endotransglucosylase/hydrolase protein 15	AT4G14130											
Probable xyloglucan endotransglucosylase/hydrolase protein 5	AT5G13870											
**PEPTIDASES**												
Xylem bark cysteine peptidase 3	AT1G09850	ER/vacuole? (TAIR/SUBA3 databases)	79.4	17.9	26.9	nd	nd	nd	12.3	1.7	6.7	35.3
Papain family cysteine protease	AT4G16190	Vacuole^(17)^	120.4	32.1	15.8	nd	nd	nd	61.8	4.7	11.2	111.0
**CYTOSKELETON PROTEINS**												
Actin 8	AT1G49240	Cytosol	78.1	8.7	19.3	17.3	2.1	**5.8**	14.7	1.7	10.0	119.0
Actin 2	AT3G18780											
Beta-6 tubulin	AT5G12250	Cytosol	15.6	4.4	15.5	nd	nd	nd	17.0	1.7	15.3	23.0
Tubulin beta-5 chain	AT1G20010	Cytosol	15.3	5.1	11.7	29.2	2.5	6.4	nd	nd	nd	nd
Tubulin beta-1 chain	AT1G75780											
**TRANSPORT**												
**Intracellular Lipid Transport**												
Trigalactosyldiacylglycerol2 (TGD2)	AT3G20320	Plastid envelope^(18)^	21.7	6.7	6.8	22.9	7.0	11.9	13.3	**1.5**	10.7	27.3
**Ion Transmembrane Transporter**												
ATPase, F0/V0 complex, subunit C protein	AT1G19910	Vacuole (TAIR/SUBA3 databases)	27.1	2.3	**5.6**	64.4	9.2	29.4	22.7	3.9	9.2	66.4
Vacuolar H^+^-pumping ATPase	AT1G75630	ER and vesicles^(19)^										
V-type proton ATPase	AT2G16510	Vacuole/endosomes^(20)^										
Autoinhibited Ca^(2+)^-ATPase, isoform 4	AT2G41560	Vacuole^(21)^	57.5	3.5	9.2	22.1	1.6	7.9	**0.5**	**0.2**	**0.7**	28.1
**Intracellular Protein Transport**												
Alpha-soluble NSF attachment protein 2	AT3G56190	Cytosol? (SUBA3)	12.0	1.5	8.3	10.3	2.6	6.5	3.8	**0.8**	**2.2**	12.8
Alpha-soluble NSF attachment protein 1	AT3G56450	ER? (SUBA3)										
N-ethylmaleimide sensitive factor	AT4G04910	Cytosol? (SUBA3)	27.1	2.3	**5.6**	64.4	9.2	29.4	22.7	3.9	9.2	66.4
ATP-Binding Cassette E2	AT4G19210	Cytosol or Membrane? (SUBA3/Uniprot)	20.5	1.8	11.4	9.9	5.0	9.1	nd	nd	nd	nd
**MISCELLANEOUS**												
Unknown protein	AT5G16550	Undetermined	999.7	208.9	397.7	151.4	42.9	113.6	112.7	81.5	44.6	26.5
HAD superfamily, subfamily IIIB acid phosphatase	AT5G44020	Unclear	155.2	7.8	121.1	9.7	7.5	36.9	nd	nd	nd	nd

a *Biological process according to MapMan. UniProt Gene Ontology and KEGG BRITE database*.

b *Protein name according to TAIR database*.

c *Gene identifier from TAIR database*.

d *Subcellular localization according to literature when available, or to TAIR, Uniprot, and SUBA3 databases. ER: endoplasmic reticulum. Ratio values below the cutoffs in one of the three experiments are in bold*.

(1) Aubert et al. (2010);

(2) Shimada et al. (2014);

(3) Gidda et al. (2016);

(4) Kim et al. (2016);

(5) De Luca and Cutler (1987);

(6) Guirimand et al. (2010);

(7) Crowell et al. (2007);

(8) Kawai et al. (2014);

(9) Wittstock and Burow (2010);

(10) Christ et al. (2013);

(11) Schenk et al. (2007);

(12) Schröder et al. (1997);

(13) Keith et al. (1991);

(14) Muday and Herrmann (1992);

(15) Iida et al. (2009);

(16) Koroleva et al. (2005);

(17) Shen et al. (2013);

(18) Awai et al. (2006);

(19) Kim H. J. et al. (2011);

(20) Zhou et al. (2016);

(21) *Geisler et al. (2000)*.

**Figure 2:**
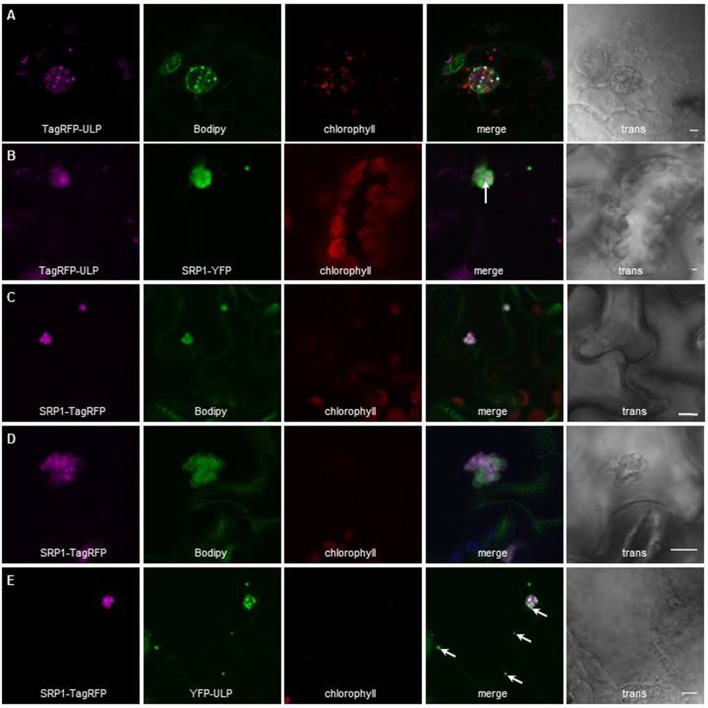
Localization of AtULP and AtSRP1-fluorescent fusions in lipid droplets. Arabidopsis cotyledons **(A,C–E)**, or *N. benthamiana* leaves **(B)** expressing TagRFP-AtULP **(A,B)** AtSRP1-YFP **(B)**, AtSRP1-TagRFP **(C–E)**, or YFP-AtULP **(E)** were visualized by confocal laser scanning microscopy. Arabidopsis plantlets were co-labeled with neutral lipid specific dye BODIPY^493/503^**(A,C,D)**. Co-labeling of AtULP or AtSRP1 targeted structures by BODIPY^493/503^ confirmed that these structures are lipid droplets. Bodipy, chlorophyll, and trans indicate Bodipy fluorescence, chlorophyll autofluorescence and transmission microscopy image respectively. Merge indicates merge of TagRFP, Bodipy and chlorophyll fluorescences **(A,C,D)** or YFP and TagRFP fluorescence **(B,E)**. Bar: 5 μm. White arrows indicate specific localization of AtULP in cells expressing both AtSRP1 and AtULP transgenes.

To further validate our proteomic approach we tested the localization of another protein highly enriched in LDs according to our proteomics data. We transiently expressed fluorescent fusions of this protein of unknown function, At5g16550, which we choose to call AtULP for Unknown Lipid droplet Protein, either alone in Arabidopsis cotyledons, or in *N. benthamiana* leaves co-transformed with *AtSRP1* fluorescent constructs. The localization of the fluorescent constructs was then observed by confocal laser scanning microscopy. In Arabidopsis cotyledons AtULP, fused to TagRFP at its N-terminal end, co-localized with cytosolic particles stained with the neutral lipid-specific fluorescent dye, BODIPY^493/503^ (MolecularProbes), suggesting that these structures are lipid rich bodies (Figure 2A). When transiently co-expressed with *AtSRP1-YFP* constructs in *N. benthamiana* leaves, the AtULP-TagRFP fluorescence was localized in the same particles as AtSRP1-YFP (Figure 2B), a protein recently shown to be targeted to LDs (Gidda et al., 2016; Kim et al., 2016). Taken together, these results demonstrate the reliability of the LD core proteome identity established here.

### Leaf LDs contain enzymes of secondary metabolite pathways involved in resistance against pathogens

Among the proteins identified here as core components of the aging leaf LD proteome we found three cytoskeleton proteins, which reflects the dynamism of LD intracellular trafficking (Welte, 2009). Such proteins were previously identified in seed (reviewed in Jolivet et al., 2013) and *Chlamydomonas reinhardtii* (Tsai et al., 2015) LDs and were recently demonstrated to participate in the recruitment and shape maintenance of LDs in zebrafish (Dutta and Kumar Sinha, 2015). It is highly probable that LDs associate to the cytoskeleton network to transit between organelles in leaves, thus allowing lipid transfer between the ER, plastids and peroxisome. Two proteins of the SNARE (Soluble N-ethylmaleimide-sensitive factor activating protein receptor) system, N-ethylmaleimide sensitive factor (NSF- At4g04910) and alpha soluble NSF attachment protein 2 (At3g56190) were also identified in the core proteome. SNARE proteins are suggested to transit from the plasma membrane or endosomes to LDs in mammalian cells and to be essential for LD growth (Boström et al., 2007). Additionally, the presence of vacuolar ATPase in the core proteome may reflect contacts between LDs and the vacuole. In yeast, LDs can be targeted to vacuoles to be degraded by a process resembling microautophagy (Vevea et al., 2015). Nevertheless, the abundance of these ATPases in leaf LDs is very low, thus their genuine association with LDs remains to be confirmed.

**Figure 3:**
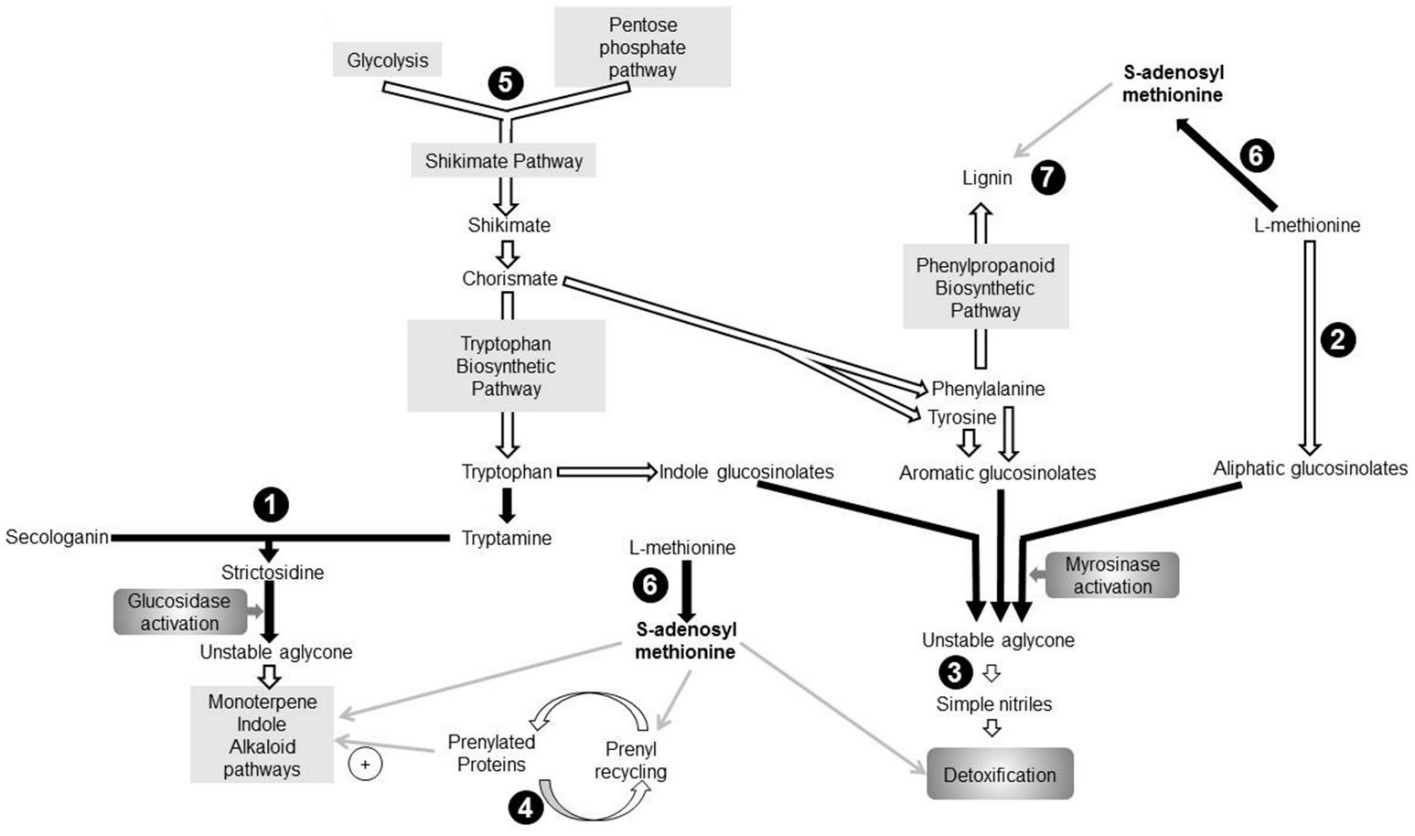
Seven proteins identified in the aging leaf lipid droplet (LD) core proteome participate to secondary metabolism pathways. Enzymes identified in leaf LDs are represented in black circles. Pathways are represented by gray rectangles. Black arrows indicate direct reactions, white arrows represent indirect reactions, and gray arrows depict involvement of one component in a pathway. (1): 3-deoxy-d-arabino-heptulosonate 7-phosphate synthase (DAHP; AT1G22410), (2): Strictosidine synthase (AT1G74010; AT1G74020), (3): Farnesylcysteine lyase (AT5G63910), (4): Methionine adenosyltransferase3 (AT2G36880), (5): 2-oxoglutarate (2OG) and Fe(II)-dependent oxygenase superfamily protein (AT2G25450), (6): Nitrile specifier protein5 (AT5G48180), (7): Peroxidase superfamily protein (Peroxidase70; AT5G64110). DMAPP, Dimethylallyl diphosphate; IPP, Isopentenyl diphosphate; MEP, Methyl-D-erythritol 4-phosphate; MIA, Monoterpenoid indole alkaloid biosynthetic pathway; PP, Diphosphate.

Surprisingly, the LD core proteome contains nine proteins involved in various secondary metabolite pathways associated with plant response to biotic stress (Figure 3). In addition to AtCLO3 and α-DOX1 involved in oxylipin metabolism (Shimada et al., 2014), we identified three proteins which directly participate in mechanisms for plant resistance to herbivores and pathogens: strictosidine synthase (STR) 2-oxoglutarate and Fe(II)-dependent oxygenase (GSL-OH) and nitrile specifier protein5 (NSP5). In addition, we found the farnesylcysteine lyase (FCLY) that contributes to cell detoxification via the recycling of prenylated proteins (Crowell et al., 2007) as well as enzymes of the amino acid metabolism: the 3-deoxy-d-arabino-heptulosonate 7-phosphate (DAHP) synthase that participates in the shikimate pathway, and a methionine adenosyltransferase 3 (SAMS3). The potential functional relevance of these proteins is discussed thereafter.

STR participates to the “nuclear time bomb” for defense against herbivory (Guirimand et al., 2010) and catalyzes the synthesis of strictosidine (St-Pierre et al., 1999), thereafter activated in an unstable aglycone (Figure 3). This aglycone is a precursor of monoterpene indole alkaloid (MIA) biosynthesis pathway (Rischer et al., 2006; Barleben et al., 2007). MIAs are critical for plant defense against biotic stress, and possess a wide range of pharmaceutical properties and applications. The subcellular localization of STR is controversial, and its exact localization in *A. thaliana* remains elusive. It is predicted to be extracellular by the SUBAtool (Hooper et al., 2014), but subcellular fractionation of *Catharanthus roseus* leaves showed that strictosidine synthase was cytoplasmic (De Luca and Cutler, 1987) whereas fluorescent microscopy pointed to a vacuolar localization (Guirimand et al., 2010). The enrichment of STR in the proteome of LDs suggests that strictosidine synthesis occurs at these organelles, the LD thus providing storage for the molecules before their subsequent activation.

The “mustard oil bomb” or glucosinolate-myrosinase defense system implies either aliphatic, aromatic or indole glucosinolates. The GSL-OH protein catalyzes one of the last steps of aliphatic glucosinolate biosynthesis (Figure 3), and the expression of the corresponding gene correlates with the accumulation of aliphatic glucosinolates in leaves (Hansen et al., 2008). Furthermore, the accumulation of 2-hydroxybut-3-enyl glucosinolate synthesized by this GSL-OH in leaves provides an increased resistance to insect herbivory. All plant GSL-OH are predicted to be cytosolic (Kawai et al., 2014). Upon biotic or abiotic stress, glucosinolates are hydrolysed into an unstable aglycone which is converted in different active products such as isothyocianates, thiocyanates or simple nitriles. NSP5 was shown to convert unstable aglycones into simple nitriles *in vitro* (Kissen and Bones, 2009; Kong et al., 2012). *A. thaliana* nitrile specifier proteins are predicted to be cytosolic (Wittstock and Burow, 2010). Here we point to a LD colocalization of an enzyme catalyzing the synthesis of glucosinolate (GSL-OH) with NSP5. This could represent a security system favoring the synthesis of simple nitrile rather than breakdown compounds with a higher toxicity. An FCLY activity was detected in membrane fraction of whole *A. thaliana* extract (Crowell et al., 2007), suggesting that it is not specifically localized in LDs but may partition between both compartments. Interestingly, a prenyl cysteine lyase has been identified in human lipoproteins (Banfi et al., 2009), suggesting that the hydrophobic environment procured by lipoproteins or LDs may provide adequate conditions for lipidated cystein recycling. Prenylated proteins are involved in response to biotic and abiotic stress (Galichet and Gruissem, 2003; Crowell and Huizinga, 2009), thus indirectly linking FCLY and plant stress response. Furthermore, some prenylated proteins also regulate the MIA biosynthetic pathway in *C. roseus* (Courdavault et al., 2009).

Our work also reveals DAHP as part of the LD proteome of aging leaves, a protein with a potential role in plant response to stress as both *A. thaliana* isoforms respond to wounding and/or pathogen attacks (Keith et al., 1991). DAHP synthases catalyze the first stage of shikimate biosynthesis which yields the three aromatic amino acids incorporated into diverse secondary metabolites, such as alkaloids, glucosinolates, or phenylpropanoid, a precursor of lignin (Maeda and Dudareva, 2012). In addition to the well-established plastidial shikimate pathway, some plants also possess cytosolic isoforms of the enzymes (Maeda and Dudareva, 2012). For example, Muday and Herrmann (1992) identified two DAHP synthase isoenzymes in *Solanum tuberosum* with cytosolic or plastidial localizations. Even though both *A. thaliana* DAHP synthases displayed a chloroplast transit peptide, it was suggested that an alternative transcription start site of transcript of DAHP synthase At4g33510 exists, possibly generating a truncated variant without N-terminal chloroplast transit peptide (Keith et al., 1991; Iida et al., 2009) that could target LDs.

SAMS3, another LD-enriched protein found in our study, catalyzes the production of S-adenosyl-L-methionine (SAM) which participates in multiple pathways (Roje, 2006), including some described hereinabove (Figure 3). (i) SAM is involved in the MIA biosynthetic pathway (Fahn et al., 1985). (ii) SAM participates in the detoxification of glucosinolate sub-products. As mentioned before, the glucosinolate catabolism generates a variety of toxic breakdown products involved in plant defense against biotic stress. In the presence of SAM, thiol methyltransferases catalyze the methylation of these compounds into less toxic products associated with airborne plant-insect signals and plant-pathogen interactions (Attieh et al., 2000, 2002). (iii) SAM is involved in the recycling of prenylated proteins: when isoprenylated proteins transit throught the ER, the prenylcysteine is methylated by an isoprenylcysteine methyltransferase with SAM as methyl donor. (iv) SAMS3 is involved in lignin biosynthesis during which SAM is a substrate for multiple methylations (Campbell and Sederoff, 1996; Shen et al., 2002). Lignin itself is synthesized from products of the shikimate pathway (Maeda and Dudareva, 2012) and deposited at the cell wall in response to stress (Le Gall et al., 2015). In *C. roseus*, the 3 SAMS isoenzymes localize in the cytoplasm and their microenvironment seems to participate in their specialization (Schröder et al., 1997). In this context, we propose that the presence of SAMS3 in LDs of aging leaves procure close proximity with other afore-mentioned enzymes thus, enabling the corresponding reactions. In other words, in senescing tissue, the LD would provide a platform where the concentration of enzymes of the secondary pathways will allow a fast and coordinated response to a variety of biotic stress. Our model thus reinforce the idea proposed by Shimada et al. (2014, 2015) for LDs and phytoalexins: during senescence, leaf LDs participate to plant defense by accumulating a mixture of compounds with antimicrobial properties or enzymes involved in the synthesis of such compounds.

### Leaf LDs are deprived of lipid metabolism enzymes

In contrast to the LD proteomes of seeds (Jolivet et al., 2013), or microalgae (Goold et al., 2015), we did not identified enzymes involved in sterol or TAG metabolism in leaf LDs. This suggests that (i) the breakdown of TAG or steryl ester does not occur at LDs in aging leaves, on the contrary to what is described in germinating seeds (Eastmond, 2006); (ii) Leaf LDs do not grow by local lipid synthesis. Instead, LD growth would occur by direct transfer of neutral lipids from the ER or by LD fusion via, for example, the SNARE system. In leaves of *AtSRP1* overexpressing lines, we observed LDs in contact with or in close proximity to, the ER membranes (see below). This suggests that leaf LDs are not isolated from the ER, but rather share a connection that would allow for a rapid transfer of molecules, such as lipids. TGD2 was the only enzyme involved in lipid metabolism identified in the core proteome of LDs. Located at the inner membrane of the plastid envelope, TGD2 is part of a transporter complex involved in lipid transfer from the ER to the plastid (Awai et al., 2006). Its presence in LDs is unlikely originating from contamination by chloroplast membranes, since we did not find any other abundant proteins from this compartment. Furthermore, TGD2 was also described in the LD proteome of *Chlamydomonas reinhardtii*, where it was proposed to originate from contact sites established between LDs and plastid envelope (Tsai et al., 2015). Interestingly we often observed similar contacts by TEM in aging leaves (Figure 1A4), supporting a genuine association of TGD2 with leaf LDs. However, additional studies are required to further substantiate and understand how this inner envelope membrane protein could be associated with cytosolic LDs.

### Aging leaf LDs are mainly composed of the caleosin AtCLO3 and the small rubber particle protein AtSRP1

In order to estimate the abundance of each protein constituting the leaf LD proteome, a TOP-3 quantitative analysis was carried out on the nine replicates of the LD fraction (Table 2). This reliable method was previously used in diverse label free quantitative proteomic approaches (Ahrné et al., 2013; Nikolovski et al., 2014). It is based on the correlation of the sum intensity of the three most intense peptides of a protein with its abundance in the sample (Silva et al., 2006). Results from this analysis showed that the two most abundant proteins of aging leaf LDs are the caleosin AtCLO3 and the small rubber particle protein AtSRP1, together constituting more than 70% of the LD protein mass. Seed LDs are described to contain several oleosins and caleosins to maintain the LD structure. However, in leaf LDs only one isoform of caleosin, AtCLO3, was detected. AtCLO3 is believed to play structural role in LDs (Chapman et al., 2012). In addition AtCLO3 has a peroxigenase activity and is involved in oxylipin biosynthesis together with α-DOX1 (Blée et al., 2014; Shimada et al., 2014). Interestingly, we found that AtCLO3 is over a 100 times more abundant than α-DOX1 in the leaf LD proteome. This suggests that only a few AtCLO3 molecules are engaged in a complex with α-DOX1, enabling the major part of AtCLO3 to play a role toward structure maintenance or other functions. AtCLO4, another caleosin, was previously reported to localize in leaf LDs (Kim Y. Y. et al., 2011), yet we did not find it enriched in our LD proteome. This may reflect that AtCLO4 is not specifically enriched in LDs but partitions with other compartments as suggested by the localization pattern of AtCLO4-GFP fusion (Kim Y. Y. et al., 2011). Alternatively, endogenous AtCLO4 may not be present in aging leaves, since it was not detected in total leaf extracts. In addition to AtCLO3, we found that AtSRP1 is highly enriched in the LD proteome of aging leaves. AtSRP1 is part of the small rubber particle family, constituted of 3 members, which was recently characterized to localize in vegetative LDs (Gidda et al., 2016; Kim et al., 2016). Interestingly, we only detected AtSRP1 in our proteome, suggesting that this protein has an unsuspected specific role in aging leaves compared to AtSRP2 and AtSRP3.

**Table 2:** Protein abundance in aging leaf LD determined by TOP3 method.

**UniProtKB ID^b^**	**GENE ID^c^**	**Protein name^d^**	**Abundance in LD estimated by TOP3 method**			**Average Abundance^e^**	**Average Protein Mass contribution (%)^f^**
			**Exp. 1**	**Exp. 2**	**Exp. 3**		
**KNOWN LIPID DROPLET PROTEINS***^a^*							
PXG3_ARATH	AT2G33380	Caleosin-related family protein (CLO3/RD20)	1.16E+08	2.35E+07	1.62E+07	5.20E+07	50.18
DOX1_ARATH	AT3G01420	Peroxidase superfamily protein (α-DOX1)	5.50E+05	2.30E+05	4.47E+04	2.75E+05	0.28
Y1736_ARATH	AT1G67360	Rubber elongation factor protein (SRPP1/LDAP1)	7.79E+07	7.87E+06	7.20E+06	3.10E+07	23.86
**BIOSYNTHESIS OF SECONDARY METABOLITES**							
Q9C9C2_ARATH	AT1G74010	Strictosidine synthase 2 (STR2)	5.18E+06	9.02E+05	6.55E+05	2.25E+06	2.06
SSL12_ARATH	AT1G74020						
PCYOX_ARATH	AT5G63910	Farnesylcysteine lyase (FCLY)	2.62E+05	2.83E+05	nd	2.05E+05	0.31
GSL_ARATH	AT2G25450	2-Oxoglutarate (2OG) and Fe(II)-dependent oxygenase superfamily protein (GSL-OH)	4.57E+05	2.06E+06	nd	1.26E+06	2.24
NSP5_ARATH	AT5G48180	Nitrile specifier protein 5 (NSP5)	9.26E+04	1.98E+05	nd	1.45E+05	0.23
C89A9_ARATH	AT3G03470	Cytochrome P450. family 87. subfamily A	7.30E+05	4.58E+05	nd	5.94E+05	0.62
CLH1_ARATH	AT1G19670	Chlorophyllase 1	1.94E+06	1.15E+06	2.94E+05	1.13E+06	1.39
**BIOSYNTHESIS OF AMINO ACIDS**							
METK3_ARATH	AT2G36880	Methionine adenosyltransferase 3 (SAMS3)	1.97E+06	3.28E+05	2.05E+05	8.35E+05	0.72
Q9SK84_ARATH	AT1G22410	3-Deoxy-d-arabino-heptulosonate 7-phosphate synthase (DHAP)	1.47E+06	2.56E+05	5.09E+04	5.92E+05	0.43
AROG_ARATH	AT4G33510						
**STRESS RESPONSE**							
ERD7_ARATH	AT2G17840	Early-responsive to dehydration 7 (ERD7)	5.92E+05	3.32E+05	3.52E+04	3.20E+05	0.35
PER70_ARATH	AT5G64110	Peroxidase superfamily protein	4.31E+05	nd	5.16E+04	2.41E+05	0.17
EXOL4_ARATH	AT5G09440	EXORDIUM like 4	nd	2.85E+05	1.54E+05	2.20E+05	0.57
**CELL WALL MODIFICATION**							
XTH4_ARATH	AT2G06850	Xyloglucan endotransglucosylase/hydrolase 4	nd	9.88E+05	2.29E+05	6.08E+05	1.43
XTH16_ARATH	AT3G23730	Probable xyloglucan endotransglucosylase/hydrolase protein 16					
XTH15_ARATH	AT4G14130	Xyloglucan endotransglucosylase/hydrolase protein 15					
XTH5_ARATH	AT5G13870	Probable xyloglucan endotransglucosylase/hydrolase protein 5					
**PEPTIDASES**							
Q0WVJ5_ARATH	AT1G09850	Xylem bark cysteine peptidase 3	1.51E+06	nd	7.19E+04	7.89E+05	0.42
Q9SUL1_ARATH	AT4G16190	Papain family cysteine protease	4.49E+05	nd	7.18E+04	2.60E+05	0.21
**CYTOSKELETON PROTEINS**							
ACT8_ARATH	AT1G49240	Actin	3.06E+07	6.72E+06	2.66E+06	1.33E+07	11.71
ACT2_ARATH	AT3G18780	Actin 2					
TBB6_ARATH	AT5G12250	Beta-6 tubulin	1.52E+06	nd	1.23E+05	8.20E+05	0.51
TBB5_ARATH	AT1G20010	Tubulin beta-5 chain	5.17E+06	5.52E+05	2.00E+05	1.98E+06	1.28
TBB1_ARATH	AT1G75780	Tubulin beta-1 chain					
**TRANSPORT**							
**Intracellular lipid transport**							
TGD2_ARATH	AT3G20320	Trigalactosyldiacylglycerol2 (TGD2)	6.67E+05	2.31E+05	4.06E+04	3.13E+05	0.29
**Ion transmembrane transporter**							
VATL2_ARATH	AT1G19910	ATPase, F0/V0 complex, subunit C protein	2.12E+05			1.09E+05	0.12
VATL4_ARATH	AT1G75630	Vacuolar H+-pumping ATPase		9.45E+04	1.90E+04		
VATL5_ARATH	AT2G16510	V-type proton ATPase					
ACA4_ARATH	AT2G41560	Autoinhibited Ca(2+)-ATPase, isoform 4	3.32E+05	1.48E+05	7.60E+03	1.62E+05	0.15
**Intracellular protein transport**							
SNAA2_ARATH	AT3G56190	Alpha-soluble NSF attachment protein 2	9.64E+05	2.55E+05	6.13E+04	4.27E+05	0.37
SNAA1_ARATH	AT3G56450	Alpha-soluble NSF attachment protein 1					
NSF_ARATH	AT4G04910	N-ethylmaleimide sensitive factor	2.12E+05	9.45E+04	1.90E+04	1.09E+05	0.12
AB2E_ARATH	AT4G19210	ATP-Binding Cassette E2	3.26E+05	6.02E+04	nd	1.93E+05	0.13
**MISCELLANEOUS**							
Q94EY7_ARATH	AT5G16550	Unknown protein	8.31E+06	6.93E+05	3.40E+05	3.12E+06	1.95
Q9FNC4_ARATH	AT5G44020	HAD superfamily, subfamily IIIB acid phosphatase	nd	1.21E+05	nd	1.21E+05	0.25

a *Biological process according to MapMan. UniProt Gene Ontology and KEGG BRITE database*.

b *Protein UniProt identifier*.

c *Gene identifier from TAIR database*.

d *Protein name according to TAIR database*.

e *Average of normalized abundance in LD estimated by TOP3 method for the three experiments*.

f *Average of protein mass contribution in LD (in percent)*.

**Figure 4:**
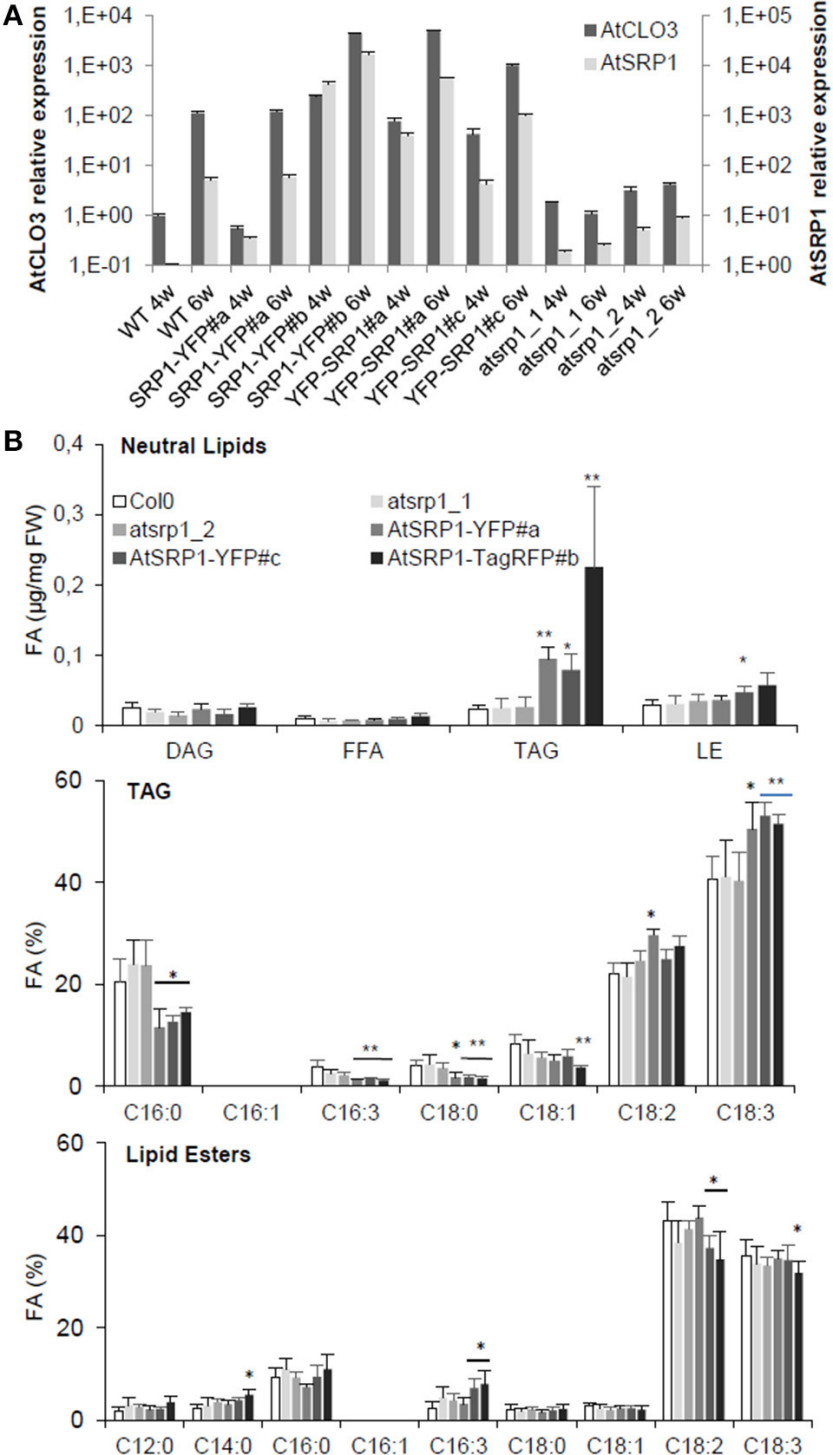
Impact of *AtSRP1* misexpression on *AtCLO3* expression and neutral lipid metabolism. **(A)** Relative quantification of *AtCLO3* and *AtSRP1* expression in wild-type (WT), *AtSRP1* overexpressors (SRP1-YFP and YFP-SRP1), and *atsrp1* knock-down (atsrp1_1 and atsrp1_2) lines. Relative expression levels of *AtSRP1* were determined by quantitative RT-PCR in 4 (4w) and 6 (6w) week old plants, and normalized to three reference genes: actin, GAPDH, and eIF4A-1. Relative expression quantities are represented related to wild type level at 4 weeks, which was set to one. Error bars represent standard deviation of technical replicates. Similar results were obtained when repeated with different plants (see Figure S8). **(B)** Neutral lipid analysis of *AtSRP1* knock-down or overexpressing leaves. Lipids from 6 week-old leaves were quantified by GC-FID after transesterification. Neutral lipid quantification (in μg of fatty acid (FA)/mg fresh weight) is represented in top panel, triacylglycerol (TAG) fatty acid composition (in % of total FA) in middle, and lipid ester fatty acid composition (in % of total FA) in down panel. Values represent average and SD from six biological replicates. Statistically significant differences from the wild type are indicated by ^*^p < 0.05 or ^**^p < 0.01 as determined by Wilcoxon's-test.

**Figure 5:**
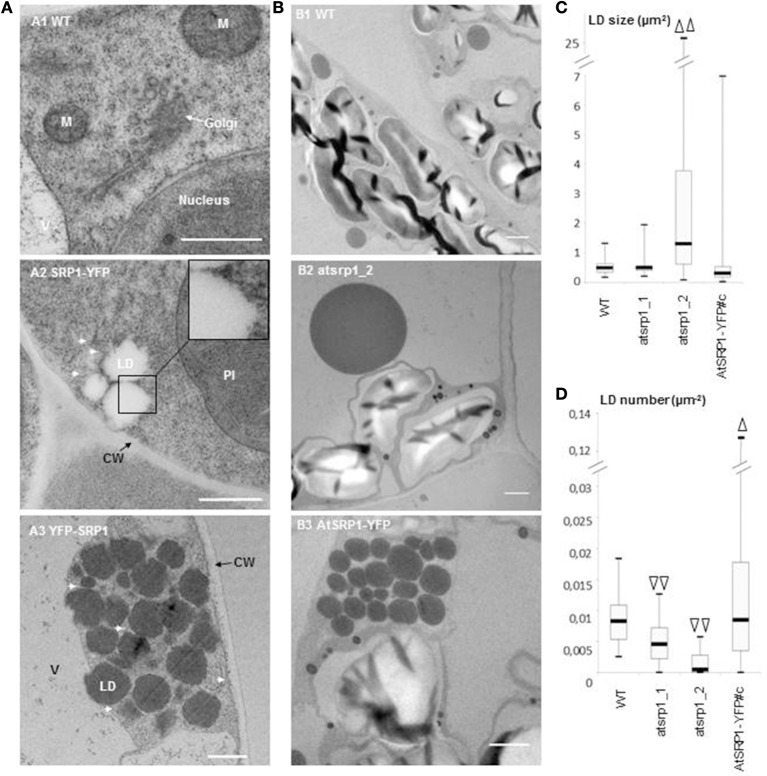
Ultrastructure study of AtSRP1 knock-down and overexpressing leaves. **(A)** Ultrastructure of lipid droplet (LD) clusters in AtSRP1 overexpressing leaves. Cryo-fixed and cryo-substituted first leaves of 6 day old wild type **(A1)** or *pUB10::AtSRP1-YFP* **(A2)** transgenic plantlets, and chemically fixed leaves of 20 day old *pUB10::YFP-AtSRP1* plantlets **(A3)** were observed by TEM. M, mitochondria; V, vacuole; CW, cell wall. For cosmetic reason, only one LD is indicated among a cluster. White arrows show endoplasmic reticulum membranes in proximity to LDs. Bar = 500 nm. **(B–D)** Ultrastructure of AtSRP1 misexpressing leaves under nitrogen starvation. Fifteen day-old plantlets were incubated on medium deprived of nitrogen for 5 days. The ultrastructure of the fourth leaf of wild type **(B1)**, *atsrp1* knock down **(B2)**, and *AtSRP1-YFP* overexpressing **(B3)** plants were observed by TEM after chemical fixation **(B)**. For each line, lipid droplet (LD) area **(C)** and LD number per cell area **(D)** were determined thanks to the ImageJ software. Arrowheads show significant differences compared to the wild-type, as determined by Student's test [*n* > 67 in **(C)** and *n* > 36 in **(D)**, ^Δ^*p* < 0.05, and ^ΔΔ^*p* < 0.01]. Measurements of LD area obtained for each individual plant are presented as box-plots in Figure S9. Bar in B = 1 μm. WT: wild-type Columbia 0, atsrp1_1 and atsrp1_2: *AtSRP1* knock-down lines, AtSRP1-YFP: *AtSRP1* overexpressing lines under *pUB10* promoter, with YFP fused at the C-term of AtSRP1.

The high content of AtSRP1 in aging leaf LDs prompted us to explore its function in these compartments. We thus generated Arabidopsis lines overexpressing AtSRP1 fused to fluorescent tags. The overexpression of the transgenes in leaves was confirmed by quantitative RT-PCR (Figure S4). We also obtained two T-DNA insertional mutants of *AtSRP1* from the ABRC. Basal transcript levels of *AtSRP1* were detected by quantitative RT-PCR in 4 and 6 week old T-DNA mutants (Figure 4A), demonstrating that they are knock-down rather than knock-out lines. Because AtCLO3 is the most abundant protein and the unique caleosin found in leaf LDs, we compared the expression levels of *AtSRP1* and *AtCLO3*, in knock-down as well as in four independent *AtSRP1* over-expressing lines. In 4 and 6 week old leaves, grown in the same conditions used for the proteomic approach, the expression of *AtCLO3* strictly follows that of *AtSRP1* (Figure 5A, Figure S5). *AtCLO3* is up-regulated in *AtSRP1* over-expressing lines, and down-regulated in *AtSRP1* knock-down lines. This indicates that *AtSRP1* is critical for the regulation of *AtCLO3*. Both caleosin and SRP proteins have been demonstrated to participate in stabilizing artificial oil bodies or inhibiting their coalescence (Chen et al., 2004; Jiang et al., 2009; Berthelot et al., 2012, 2014). Their high content in LDs of aging leaves, as well as the co-regulation of *AtCLO3* and *AtSRP1* expression, suggest that they both play fundamental role in stabilizing leaf LD structure and that the maintenance of a strict stoichiometry between both proteins may be determinant to preserve the LD structure.

### Different vegetative LDs co-exist in cotyledons and mature leaves

Next, we assayed the localization of fluorescent AtSRP1 constructs using confocal laser scanning microscopy. In agreement with Gidda et al. (2016) and Kim et al. (2016), AtSRP1-TagRFP co-localized with cytosolic mobile particles stained with the neutral lipid-specific fluorescent dye BODIPY^493/503^, suggesting that AtSRP1 localizes to lipid rich bodies (Figure 2C). A similar localization was observed with AtSRP1 constructs fused to YFP or TagRFP either at the C- or N-terminal of the protein. We further observed larger BODIPY-stained AtSRP1 structures present in cotyledon (Figure 2D and Figure S6) and noticed that (i) these large lipid rich bodies are in fact lipid droplet aggregates, and that (ii) the TagRFP fluorescence seemed restricted to the periphery of the lipid droplet. These aggregates of LDs were not observed in wild-type plants, implying that the overexpression of *AtSRP1* induces the neo-formation of LD clusters in standard growth conditions.

Similarly to AtSRP1, AtULP, an uncharacterized LD-enriched protein (Figure 2), was visualized in clusters of LDs in agro-infiltrated leaves of *N. benthamiana*. When co-expressed in the same cell, both targeted the same LD clusters, but not exactly at the same area of the clusters, suggesting that AtULP and AtSRP1 are not systematically targeted to the same LDs (Figure 2B). This difference was even more striking when Arabidopsis cotyledons overexpressing *AtSRP1-TagRFP* were transiently agro-infiltrated with YFP-AtULP (Figure 2E): YFP-AtULP fluorescence was observed in LD clusters tagged with AtSRP1-TagRFP, but also in some isolated LDs of the same cell untagged by AtSRP1-TagRFP. This suggests that diverse populations of LDs, with differential protein compositions, could co-exist in cotyledons and leaves. In cotyledons, these diverse populations could correspond to a concomitant mix of “seed” LDs derived from the embryo with neo-formed “vegetative” LDs. Nevertheless, their observation in mature leaves of *N. benthamiana* strongly supports our upraising idea that various specialized LDs coexist in vegetative tissues.

### AtSRP1 overexpression in leaves induces the formation of new LDs organized in clusters in contact or in continuity with the ER

To gain insight into the LD ultrastructure, we performed transmission electron microscopy (TEM) after cryo-fixation of *AtSRP1* overexpressing Arabidopsis leaves. This analysis showed an accumulation of round-shaped cytosolic aggregates, which were never observed in wild-type leaves (Figure 5A2). To confirm the nature of these clusters, immunogold labeling with an antibody specific to the GFP/YFP protein was performed on cryo-fixed roots of a *YFP-AtSRP1* overexpressing line. Labeling was restricted to these cytosolic aggregates (Figure S7), while no such specific labeling was observed in control experiments where anti-GFP/YFP antibody was omitted. Therefore, our data indicate that the YFP-AtSRP1 protein is specifically associated with these aggregates. These structures were not surrounded by a typical bilayer membrane but a monolayer (insert Figure 5A2), suggesting that they are not vesicles or small vacuoles. Similar clusters homogeneously stained by osmium tetroxide were observed in chemically fixed leaves of *YFP-AtSRP1* overexpressing lines (Figure 5A3). The colocalization of AtSRP1-TagRFP protein with the neutral lipid specific dye BODIPY^493/503^ (Figure 2), together with the homogeneous staining of the aggregates by osmium tetroxide when traditional chemical fixation was used, confirmed that these structures are filled with neutral lipids. Moreover, these data confirm that the overexpression of *AtSRP1* is responsible for the formation of such LD clusters.

**Figure 6:**
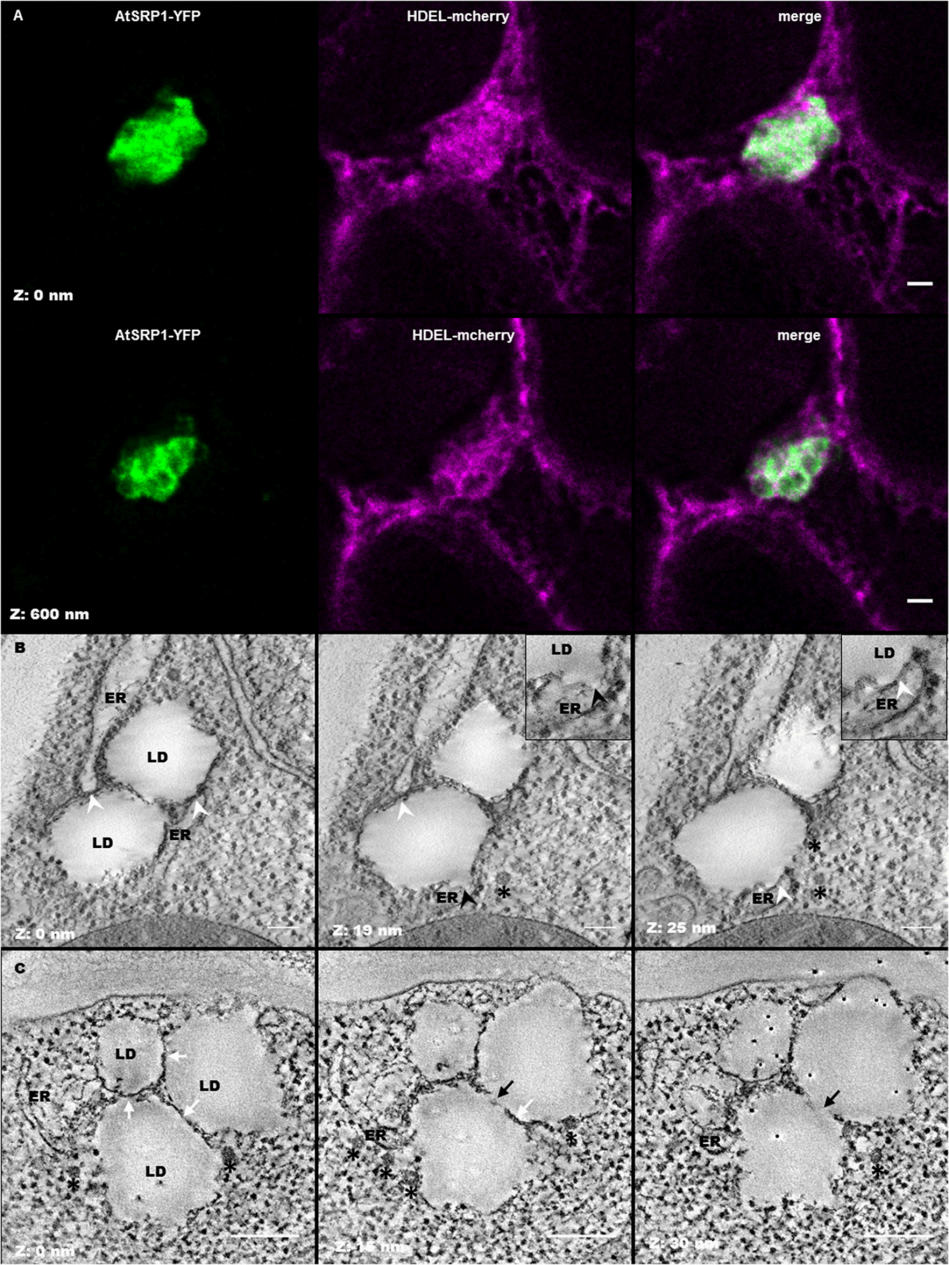
Leaf lipid droplets (LDs) are in close proximity with the endoplasmic reticulum (ER). **(A)** Fluorescent co-labeling of leaf LDs and the ER. *N. benthamiana* leaves were transiently co-transformed with AtSRP1-YFP and HDEL-mCherry constructs labeling LDs and ER membranes respectively. Deconvolution after confocal imaging of epidermal cells demonstrates multiple contact sites between LDs and the ER network, and AtSRP1-YFP localization at the LD periphery. Merge indicates overlap of YFP (green) and mCherry (magenta) fluorescences. Bar: 2 μm. **(B,C)** Electron tomography of leaf LDs in AtSRP1 overexpressing Arabidopsis plantlets with single **(B)** and dual **(C)** axis acquisition. White arrowheads indicate LD/ER contacts. Black arrowhead indicates the ER mono-leaflet at the LD budding point. White arrows indicate apposition of two LDs, black arrows indicate direct continuity between two LDs. Black asterisks are positioned below vesicle-like structures. Black points with white halo in last panel are gold fiducials used for alignment steps during tomogram. Bar: 200 nm.

LD clusters were often observed in the close vicinity of the ER membranes (Figures 5A2,A3). In order to obtain a better vision of the position of LDs in the ER network, *N. benthamiana* leaves were transiently co-transformed with *pUB10::AtSRP1-YFP* and a fluorescent marker of the ER, HDEL-mCherry (Nelson et al., 2007). As shown in Figure 6A, the transient expression of *AtSRP1* in tobacco leaves was sufficient to induce the formation of LD clusters similar to that observed in stably transformed Arabidopsis. These LD clusters were mobile, likely following cytosolic or ER streaming (Ueda et al., 2010). YFP tagged LD clusters appeared not only in contact to mCherry fluorescent ER membranes, but even trapped in the ER network, showing a multitude of contact sites between LDs and ER membranes (Figure 6A and Movie S1). Unfortunately, the confocal microscopy resolution is not sufficient to resolve the AtSRP1-ER localization and determine if AtSRP1 is exclusively present in LD periphery or also at ER-LD biogenesis sites. However, results of our proteomic approach, as well as of our IEM experiment (Figure S7) clearly argue for a preferential, if not exclusive, localization of AtSRP1 at LDs. Chapman et al. (2012) proposed that in non-seed tissues LDs originate from small LDs formed all along the ER, “pinched off form the ER, and then fused to form larger droplets.” However, the cluster organization of LDs in *AtSRP1* overexpressing lines rather suggests that LDs are formed at restricted, specialized areas of the ER.

As emphasized by Lersten et al. (2006) as well as Xu and Shanklin (2016), the biology and structure of LDs in leaf cells still remains to be examined. While one could argue that cotyledon and leaf LDs are similar organelles and that LDs have already been described in chemically fixed cotyledons (Frey-Wyssling et al., 1963; Wanner and Theimer, 1978; Wanner et al., 1981), the results of our proteomic analysis demonstrated that leaf LDs have a protein composition totally different from that of seed/cotyledon LDs, suggesting a divergence in function and possibly in structure. The ability to cryo-fix leaves was crucial to fix instantaneously the sample in a near to native state and optimize ultrastructure conservation. Aging leaves, accumulating LDs, are difficult to cryo-fix but young leaves only contain minor quantity of LDs. We thus took advantage of the induction of LD formation in young leaves of our *AtSRP1* overexpressing plants to deeply decipher the leaf LD ultrastructure using an electron tomography of LDs in leaves of 6 day old plantlets (Figure 6B and Movie S2). Our analysis showed several ER membranes closely opposed to each LD, in a conformation excluding ribosomes from these sites. Given that the exclusion of ribosomes from the ER membrane is, with the proximity of membranes, “one defining feature for membrane contact sites” (Pérez-Sancho et al., 2016; Phillips and Voeltz, 2016), this suggests that in leaf, LDs and ER share domains reminiscent of membrane contact sites, as illustrated for example in *Caenorhabditis elegans* (Xu et al., 2012). In addition, a direct continuity between the ER membrane and the LD was observed in some sections (Black arrows in Figure 6B), with the inner leaflet of the ER membrane still separating the LD from the ER lumen. This site, representing a specialized ER domain for LD formation, is highly restricted, with a thickness of only a few tens of nm. We propose that this connection, together with multiple contact sites allow the transit of metabolites between the two compartments, in particular the LD accumulation of TAGs synthesized at the ER membrane, as previously demonstrated in yeast and mammalian cells (Jacquier et al., 2011; Kassan et al., 2013; Choudhary et al., 2015). Given the numerous contact sites observed between LDs and the ER in *AtSRP1* overexpressing leaves, one can wonder why no ER protein originating from ER remnants were identified in the LD fraction. It probably reflects that ER-LD connections in wild-type plants are highly labile and dynamic, which is indispensable to allow LD intracellular motion (Welte, 2009). LDs of a same cluster seemed also appressed to each other rather than simply side by side. By electron tomography of a restricted volume, interconnections between some LDs could be observed (Figure 6C), suggesting either that newly formed LDs could bud from pre-existing one or that when a LD reaches its optimal size, the formation of a new one is induced at the exact same site of the ER. The first LD does not detach from the following one, leading to the formation of these clusters of interconnected LDs. Yet, the existence of such an interconnection between two LDs in wild-type leaf remains to be proven. Finally, multiple COP-like vesicles were observed by electron tomography in the vicinity of the LD clusters (black asterisks in Figures 6B,C). The identity of these vesicles remains elusive, however, they could participate in the LD biogenesis, similarly to the model proposed by Kalantari et al. (2010) for animal cells where “lipid droplets form in close association with ER membrane seeded from a COPI vesicle.”

### AtSRP1 regulates the biogenesis and the size of LDs in induced senescent leaves

We next evaluated the role of AtSRP1 on the biogenesis and ultrastructure of LDs under conditions inducing their formation. In these experiments, plantlets were submitted to nitrogen starvation in order to induce precocious senescence (Balazadeh et al., 2014). As expected, nitrogen starvation induced a scattered LD formation in wild type plants (Figure 5B) with an average of 0.009 ± 0.004 μm^−2^ LD per cell area (corresponding roughly to 3.3 LDs per cell section) while LDs were scarcely observable under control conditions. The average area of a single LD was 0.51 ± 0.20 μm^2^, with no LD larger than 1.3 μm^2^ (Figure 5C, Figure S9). Moreover, 15% of wild type cells contained clusters of LD, with clusters composed of a maximum of 4 LDs. Under nitrogen starvation conditions, the number of LDs per cell area in *AtSRP1* overexpressing line was significantly increased compared to the wild type (Figure 5D), with an average of 0.017 ± 0.003 μm^−2^ LDs per cell area. In addition, LDs were distributed in 50% of the cases in clusters of 2–35 LDs. The LD size in *AtSRP1* overexpressing line was heterogeneous, ranging from 0.026 to more than 6 μm^2^; yet this was not statistically significantly different from the wild type (Figure 5C, Figure S9). The effect of nitrogen starvation was also tested on the two *atsrp1* knock-down mutants where it also induced LD formation but significantly (*p* < 0.01) less than in the wild type. Furthermore, the LD size was highly heterogeneous and significantly bigger in one knock-down line compared to the wild-type (Figures 5B,D and Figure S9), with some very large LDs (up to 25 μm^2^). It seems that when knock-down *atsrp1* lines are submitted to conditions that should induce LD formation, no or few new LDs are generated, and molecules usually sequestered in newly formed LDs accumulate in pre-existing LDs thus leading to abnormally large LDs. Altogether, these results indicate that SRP1 is a key element in (1) preventing the coalescence of LDs, (2) regulating their size, and (3) inducing their neo-formation in senescent leaves.

### AtSRP1 induces accumulation of TAG enriched in C18:3 in aging leaves

The induced neo-formation of LDs in *AtSRP1* overexpressing leaves, suggests that AtSRP1 is able to mobilize components required for the formation of LDs, including structural proteins and neutral lipids. Gidda et al. (2016) described an increase of neutral lipid content in *AtSRP1* overexpressing plantlets grown for 15 days on Murashige and Skoog plates, with an enrichment of C18:2 and C18:3, and no perturbation of the lipid composition of knock-down lines. In order to better evaluate the role of AtSRP1 under conditions that naturally induce AtSRP1 expression and neutral lipid accumulation in LDs, we analyzed the neutral lipid composition of 6-week old senescent *AtSRP1* overexpressing or knock-down leaves (Figure 4B). The lipid composition of knock-down lines was not perturbed, but *AtSRP1* overexpressing lines accumulated more neutral lipids in leaves than the wild type (Figure 4B). Even though lipid esters are the most abundant neutral lipids in LDs of aging leaves (Figure 1B), the overexpression of *AtSRP1* specifically led to an increase of the TAG and not of the lipid ester content. In the three independent overexpressing lines, TAGs were more than three times more abundant than in the wild-type. In addition, overexpression of *AtSRP1* modified the TAG fatty acid composition with a significant enrichment in C18:3 acyl moieties, mainly at the expense of C16:0. By contrast, the fatty acid composition of lipid esters was only slightly modified and the fatty acid composition of galactolipids and phospholipids were similar in the wild type and in overexpressing lines (Figure S8). These data show that the overexpression of *AtSRP1* does not impact fatty acid metabolic pathways. Further, our results suggest that the significant enrichment in C18:3 acyl moieties of TAG does not originate from an imbalance in the C18:3 homeostasis but may rather originate from recycling of thylakoid galactolipids (that are rich in C18:3 and relatively poor in C16:0). The capacity of the cell to form new LDs seems to limit the recycling of acyl groups into TAGs. When AtSRP1 induces LD biogenesis, TAG synthesis is no more limited and TAG leaf content increases. An increase in TAG content was similarly described in LEC2 and/or SEIPIN overexpressing plants (Cai et al., 2015). LEC2 is a seed specific transcription factor that promotes the accumulation of TAG by stimulating the expression of seed-specific genes. AtSEIPINs are human lipodystrophy orthologs, which localize in discrete ER subdomains and promote LD biogenesis. In LEC2 and AtSEIPIN overexpressing plants, the TAG fatty acid composition is modified showing an increase of C16:0 and a decrease of C18:3 contents. C16:0 acyls are enriched in the ER, suggesting that both AtSEIPIN and LEC2 promote the accumulation of ER-derived acyl groups into TAGs. In contrast, AtSRP1 seems to induce a different mechanism which would rather promote the recycling of plastid lipids for TAG synthesis. However, additional evidences are needed to support this hypothesis. It is worth noting that, in aging leaves, LDs were predominantly observed in close proximity to plastids (more than 85% of the observed LDs were next to the plastid envelope). This further suggests a possible transfer of metabolites from the plastids to the LDs during senescence, as already suggested for plastids and ER (reviewed in Mehrshahi et al., 2014), or for plastid and LDs in *C. reinhardtii* (Goodson et al., 2011).

## Conclusion

Altogether, our fine microscopic analyses demonstrated that AtSRP1 plays a dual structural role by inducing LD formation and controlling LD size. Indeed, when LD formation is induced in *atsrp1* knock-down lines, striking large LDs are generated. Conversely, in *AtSRP1* overexpressing lines LD biogenesis is exacerbated, leading to the formation of numerous LDs that stay aggregated in clusters but do not coalesce in one large LD. These LDs stay in close proximity with the ER, being wrapped within its network, and, as illustrated by tomography, can share direct connections with the ER lumen as well as with other LDs. A tight regulation of LD size and distribution is most probably crucial for the optimal functioning of the LDs. Our proteomics data show that leaf LDs are abundantly associated with proteins of the cytoskeleton, highlighting the importance of intracellular LD motion for their functions (Welte, 2009). Large LDs or aggregation of LDs in clusters most probably impair fast cell dynamic movements as well as LD exchanges with other organelles. In addition, we show that AtSRP1 overexpression enhances TAG synthesis in a different way to that described in cotyledons with SEIPIN proteins and suggest a possible involvement of plastid lipid metabolism in LD biogenesis of senescent leaves. Because SEIPINs co-localized with LDs and the ER, and influenced the structure of the ER, Cai et al. (2015) hypothesized that “SEIPINs participate in the organization of ER subdomains that are devoted to (…) LD formation.” It is possible that once SEIPINs have reorganized the ER in specialized subdomains dedicated to LD formation, AtSRP1 is recruited to induce the formation of new LDs at these subdomains. Furthermore, AtSRP1 could maintain a correct radius curvature of the monolayer surrounding the LD, therefore controlling its size. Our tomographic study demonstrates physical connections between LDs of a same cluster and between LDs and the ER in *AtSRP1* overexpressing leaves. These connections may also exist in wild type leaves. However, given the involvement of LDs in intracellular movement and exchange, it is improbable that these connections permanently exist in the wild type. The fission of organelles, for example of coat protein (COP)II vesicles from the ER, or of the Golgi vesicles, necessitates the intervention of specialized proteins (Brown et al., 2008; Fujimoto and Tsutsumi, 2014). In the case of LDs, it is unclear if they systematically dissociate from the ER membrane and if this detachment is spontaneous or supported by specific proteins. The separation of LDs from the ER (and from other LDs) seems perturbed by the overexpression of AtSRP1, therefore indicating that this dissociation is not spontaneous. The unusual accumulation of AtSRP1 at the LD surface may impair the membrane layer fusion process necessary for LD detachment, or the proteins responsible for the LD closing and detachment process may not be enough represented in the overexpressing lines to separate all the neo-formed LDs from the ER.

Results from proteomic study presented here show that AtCLO3 and AtSRP1 are the only structural proteins present in the proteome of leaf LDs. What are the respective roles of AtSRP1 and AtCLO3, the two most abundant proteins in leaf LDs, in the maintenance of LD ultrastructure? How is SRP1 recruited to the ER? Do SEIPIN proteins interact with AtSRP1 and when? Are there any additional partners needed for the dissociation of the LDs from the ER? What is the exact chronology of the diverse events responsible for the LD biogenesis in leaves? In this purpose, the availability of AtSRP1, AtCLO3 and SEIPINs overexpressing lines, as well as the ability to cryofix plant tissues and to perform a correlative light electron microscopy approach, represent valuable tools to better understand events of the LD biogenesis in non-seed tissue.

## Accession numbers

Sequence data from this article can be found in the Arabidopsis Genome Initiative databases under the following accession numbers: *AtSRP1*: At1g67360; *AtCLO3*: At2g33380; *AtULP*: At5g16550; *actin*: At1g49240; *GAPDH*: At1g13440; *eIF4A-1*: At3g13920.

## Author contributions

LB, FI, DC, NE, and CB performed most of the experiments; LB, LF, SC, and CB supervised the experiments; KT, SP, SC, and NE provided technical assistance to LB, FI, and CB; LB, FI, JB, and CB designed the experiments and analyzed the data; CB conceived and supervised the project. FI and CB wrote the article with contributions of all the authors.

### Conflict of interest statement

The authors declare that the research was conducted in the absence of any commercial or financial relationships that could be construed as a potential conflict of interest.
