# Supplementary material for: Proteomic Analysis of Lipid Droplets from Arabidopsis Aging Leaves Brings New Insight into Their Biogenesis and Functions
Source: Front Plant Sci. 2017 May 29;8:894. doi: 10.3389/fpls.2017.00894 (PMC5447075; doi:10.3389/fpls.2017.00894)
Supplement: Supplementary file 10 [file Table1.PDF]

Primer sequences		
	**name**	**sequence 5' to 3'**
Cloning		
	srp1atgf	CACCATGGAGACAGAGAAGAAAAATAGC
	srp1-r	CTCCGAATCAGACGATGATTTATC
	srp1stop-r	CTACTCCGAATCAGACGATG
	Mut ULP forw	CACGATCGGTTCGATTCTGTTTCCAGATTCG
	Mut ULP rev	CGAATCTGGAAACAGAATCGAACCGATCGTG
T-DNA mutant genotyping		
	SRP1_1LP	ATCGACAGAAAACATGGATGG
	SRP1_1RP	CTTTGGGACCACCGGTACGAGCC
qPCR		
	qSRP1f	AGATCACAAGGTGGGTGAAG
	qSRP1r	TGGGACCACCGGTACGAGCC
	qRD20f	AATGGCAATCGATCCTTTTG
	qRD20r	AAACCATCTTCGTCCTTAGCAA
	qGAPDH-f	TTGGTGACAACAGGTCAAGCA
	qGAPDH-r	AAACTTGTCGCTCAATGCAATC
	qACT	CCGAGCAGCATGAAGATTAAG
	qACT	CATACTCTGCCTTAGAGATCCACA
	qeIF4A-1	CTGATTTTGACCCGTCGTCT
	qeIF4A-1	AAGACAAACAACAAAGCCGAAT

Table S1 : Sequences of the primers used in this study.
