# Supplementary material for: Proteomic Analysis of Lipid Droplets from Arabidopsis Aging Leaves Brings New Insight into Their Biogenesis and Functions
Source: Front Plant Sci. 2017 May 29;8:894. doi: 10.3389/fpls.2017.00894 (PMC5447075; doi:10.3389/fpls.2017.00894)
Supplement: Supplementary file 12 [file Table3.PDF]

Accession number	Name / functional information	Abundance in LD	Abundance in PG
**At4g04020**	**AtPGL35 / FBN1a** Stress tolerance	1,09E+07	2,07E+07
**At3g23400**	**AtPGL30.4 / FBN4** Plastoglobule development, stress tolerance	4,47E+07	1,08E+08
**At4g22240**	**AtPGL33 / FBN1b** Stress tolerance	2,08E+07	7,73E+07
**At2g35490**	**AtPGL40 / FBN2** Stress tolerance	7,67E+06	1,30E+07
**At3g58010**	**AtPGL34 / FBN7a**	2,54E+05	1,95E+06
**At2g42130**	**AtPGL30 / FBN7b**	2,09E+05	6,12E+05
**At2g46910**	**AtPGL31 / FBN8**	1,91E+05	7,14E+05
**At5g05200**	**ABC1 kinase 9**	3,89E+05	1,26E+06
**At4g31390**	**ABC1 kinase 1 / AtACDO1** Photooxidative stress tolerance	2,48E+05	6,74E+05
**At1g79600**	**ABC1 kinase 3**	5,16E+05	2,14E+05
**At3g24190**	**ABC1 kinase 6**	n.d.	n.d.
**At1g71810**	**ABC1 kinase 5**	2,32E+05	1,39E+05
**At3g07700**	**ABC1 kinase 7**	7,22E+04	6,89E+03
**At4g19170**	**Carotenoid cleavage dioxygenase (AtCDD4)** Carotenoid catabolism	9,64E+05	4,49E+06
**At4g32770**	**tocopherol cyclase (VTE1)** Vitamine E biosynthesis	8,22E+05	3,18E+06
**At5g08740**	**NAD(P)H dehydrogenase C1 (NDC1)** Phylloquinone synthesis, plastoquinone reduction	8,19E+05	3,59E+06
**At1g54570**	**PES 1** Phytyl ester synthesis	5,52E+05	1,65E+06
**At3g26840**	**PES 2** Phytyl ester synthesis	1,93E+05	7,80E+05
**At1g78140**	Protein with Methyltransferase type 11 domain - 1	5,19E+05	1,69E+06
**At2g41040**	Protein with Methyltransferase type 11 domain - 2	5,82E+05	8,45E+05
**At1g32220**	Protein with NAD-dependent epimerase/dehydratase domain	6,78E+05	3,17E+06
**At1g06690**	Protein with aldo-keto reductase domain	3,12E+05	6,03E+05
**At4g39730**	Lipase/lipooxygenase, PLAT/LH2 family protein	9,93E+05	1,61E+05
**At1g73750**	Protein with a/b hydrolase domain	n.d.	n.d.
**At5g41120**	Esterase/lipase/thioesterase family protein	1,51E+04	1,32E+05
**At5g42650**	**Allene oxide synthase (AOS)** Jasmonic acid biosynthesis	9,01E+05	2,79E+06
**At2g21330**	**Fructose-bisphosphate aldolase -1**	6,57E+06	1,54E+07
**At4g38970**	**Fructose-bisphosphate aldolase -2**	3,96E+06	1,15E+07
**At2g01140**	**Putative fructose-bisphosphate aldolase -3**	3,92E+05	1,84E+06
**At3g10130**	Protein with SOUL heme-binding domain	4,90E+05	1,81E+06
**At2g34460**	NAD(P)-binding Rossmann-fold superfamily protein	5,46E+05	3,54E+06
**At4g13200**	Unknown protein - 1	4,32E+05	1,66E+06
**At3g43540**	Unknown protein - 2 (DUF 1350)	6,56E+05	1,40E+06
**At3g27110**	Protein with peptidase M48 domain	5,13E+04	3,03E+05

Table S3 : Comparison of plastoglobule protein abundance between plastoglobule (PG) and lipid droplet (LD) fractions from 6 week old leaves. Abundances are the average of normalized abundances in three replicates (experiment #c), quantified by label free and corrected according to TOP3 algorythm. n.d. : not detected.
