# Supplementary material for: Proteomic Analysis of Lipid Droplets from Arabidopsis Aging Leaves Brings New Insight into Their Biogenesis and Functions
Source: Front Plant Sci. 2017 May 29;8:894. doi: 10.3389/fpls.2017.00894 (PMC5447075; doi:10.3389/fpls.2017.00894)
Supplement: Supplementary file 13 [file Table4.PDF]

PROCESS [a]	UniProtKB ID [b]	GENE ID [c]	PROTEIN NAME [d]	Location	Exp. A LD/S	LD/Mb	LD/TL (>6)	Exp. B LD/S
**[SUBCELLULAR LOCATION] Plastid, chloroplast**								
	ACCD_ARATH	ATCG00500	Acetyl-CoA carboxylase carboxyl transferase subunit beta	plastid	31,27	12,72	29,17	nd
	PAO_ARATH	AT3G44880	Pheophorbide a oxygenase family protein with Rieske [2Fe-2S] domain	plastid	42,70	16,28	13,01	55,57
	EBFC2_ARATH<br>EBFC1_ARATH	AT2G24020<br>AT4G30620	Uncharacterised BCR, YbaB family COG0718	plastid	7,23	16,70	6,02	23,23
	TRPD_ARATH	AT5G17990	Tryptophan biosynthesis 1	plastid	8,77	2,99	7,85	20,12
	SPPA1_ARATH	AT1G73990	Signal peptide peptidase	plastid	131,70	34,89	23,88	51,03

Table S4: Plastid proteins manually removed from the lipid droplet (LD) core proteome.

a: Biological process according to MapMan, UniProt Gene Ontology and KEGG BRITE database

b: Protein UniProt identifier

c: Gene identifier from TAIR database

d: Protein name according to TAIR database

Mb: membrane, S: soluble, TL: total leaf, PG: plastoglobule

Enrichment ratios						Abundance in LD estimated by TOP3 method									Average Abundance
		Exp. C				Exp. A			Exp. B			Exp. C			
LD/Mb	LD/TL (>6)	LD/S	LD/Mb	LD/TL (>6)	LD/PG (>4)	Average	LD_SD	%	Average	LD_SD	%	Average	LD_SD	%	
nd	nd	11,65	2,12	9,10	22,32	**5,33E+05**	*8,84E+04*		nd	nd		**4,65E+04**	8,18E+03		2,90E+05
9,17	12,01	24,27	4,42	***3,77***	9,15	**2,04E+06**	*3,05E+05*		**1,31E+06**	4,34E+04		**1,76E+05**	2,74E+04		1,17E+06
5,82	8,45	7,38	3,83	***2,86***	53,90	**1,52E+06**	*2,08E+05*		**1,80E+05**	4,77E+04		**3,99E+04**	7,61E+03		5,79E+05
10,76	9,62	6,61	2,91	***2,56***	11,31	**1,87E+05**	*4,55E+04*		**2,57E+05**	6,61E+03		**3,61E+04**	1,29E+04		1,60E+05
6,44	11,46	24,63	6,35	10,78	13,12	**4,53E+05**	*1,05E+05*		**1,89E+05**	2,31E+04		**4,09E+04**	1,38E+04		2,28E+05
