# Supplementary material for: Proteomic Analysis of Lipid Droplets from Arabidopsis Aging Leaves Brings New Insight into Their Biogenesis and Functions
Source: Front Plant Sci. 2017 May 29;8:894. doi: 10.3389/fpls.2017.00894 (PMC5447075; doi:10.3389/fpls.2017.00894)
Supplement: Supplementary file 16 [file DataSheet1.PDF]

Appendix S1 : Experimental procedures.

Sample preparation for Label Free analysis

Protein sample were solubilized in Laemlli buffer and 10 µg of each fraction were deposited onto a 10% acrylamide SDS gel. Migration was stopped after samples entered the resolving gel and proteins were visualized by Colloidal Blue staining. Samples were relatively quantified by densitometry. Each SDS-PAGE band was cut into 1 mm x 1 mm gel pieces. Gel pieces were destained in 25 mM ammonium bicarbonate 50% ACN, rinsed twice in ultrapure water and shrunk in ACN for 10 min. After ACN removal, gel pieces were dried at room temperature, covered with the trypsin solution (10 ng/µL in 40 mM NH4HCO3 and 10% ACN), rehydrated at 4°C for 10 min, and finally incubated overnight at 37°C. Gel pieces were then incubated for 15 min in 40 mM NH4HCO3 and 10% ACN at room temperature. The supernatant was collected, and an H2O/ACN/HCOOH (47.5:47.5:5) extraction solution was added onto gel pieces for 15 min. The extraction step was repeated twice. Supernatants were dried in a vacuum centrifuge and were resuspended in 0.1%HCOOH so as to get similar peptide concentration based on protein quantities estimated by densitometry. Samples were stored at -20°C.

nLC-MS/MS analysis

Peptide mixture was analyzed on a Ultimate 3000 nanoLC system (Dionex, Amsterdam, The Netherlands) coupled to a LTQ-Orbitrap XL mass spectrometer (Thermo Fisher Scientific, San Jose, CA). Ten microliters of peptide digests were loaded onto a 300-µm-inner diameter x 5-mm C18 PepMapTM trap column (LC Packings) at a flow rate of 20 µL/min. The peptides were eluted from the trap column onto an analytical 75-mm id x 15-cm C18 Pep-Map column (LC Packings) with a 2–40% linear gradient of solvent B in 108 min (solvent A was 0.1% formic acid in 5% ACN, and solvent B was 0.1% formic acid in 80% ACN). The separation flow rate was set at 200 nL/min. The mass spectrometer operated in positive ion mode at a 1.8-kV needle voltage. Data were acquired using Xcalibur 2.2 software in a data-dependent mode. MS scans (m/z 300-1700) were recorded at a resolution of R = 60000 (@ m/z 400) and an AGC target of 5 x 105 ions collected within 500 ms. Dynamic exclusion was et to 30 s and top 6 ions were selected from fragmentation in Collision Induced Dissociation mode. Ion trap MS/MS scans with a target value of 1 x 104 ions were collected with a maximum fill time of 200 ms. Additionally, only +2 and +3 charged ions were selected for fragmentation. Others settings were as follows: no sheath nor auxiliary gas flow, heated capillary temperature, 200°C; normalized CID collision energy of 35% and an isolation width of 2 m/z.

Database search and results processing

Data were searched by SEQUEST through Proteome Discoverer 1.4 (Thermo Fisher Scientific Inc.) against the TAIR (The Arabidopsis Information Resource) protein database (version 10; 32785 entries). Spectra from peptides higher than 5000 Da or lower than 350 Da were rejected. The search parameters were as follows: mass accuracy of the monoisotopic peptide precursor and peptide fragments was set to 10 ppm and 0.6 Da, respectively. Only b- and y-ions were considered for mass calculation. Oxidation of methionines (+16 Da), propionamide (+71 Da) and carbamidomethylation of cysteines (+57 Da) were considered as variable modifications. Two missed trypsin cleavages were allowed. Peptide validation was performed using Percolator algorithm (Käll *et al.*, 2007) and only “high confidence” peptides were retained corresponding to a 1% False Positive Rate at peptide level.

Label-Free Quantitative Data Analysis

Label-Free quantitation was performed based on XIC (Extracted Ion Current) obtained in MS1. Raw LC-MS/MS data were imported in Progenesis QI 2.0 (Nonlinear Dynamics Ltd, Newcastle, UK). Data processing includes the following steps: Features detection, Features alignment across the samples and Volume integration for 2-6 charge-state ions. Identified proteins/peptides were exported from proteome Discoverer 1.4 as XLSX files. Once pick picking conducted in Progenesis, database search reports were directly imported in Progenesis. Protein

abundancies were calculated and normalized based on the median ratio across all the feature (i.e. detected ions) abundances, and considering one experiment as the reference.

Käll, L., Canterbury, J.D., Weston, J., Noble, W.S. and MacCoss, M.J. (2007) Semi-supervised learning for peptide identification from shotgun proteomics datasets. *Nat. Methods*, **4**, 923–925.

Silva, J.C., Gorenstein, M.V., Li, G.-Z., Vissers, J.P.C. and Geromanos, S.J. (2006) Absolute quantification of proteins by LCMSE: a virtue of parallel MS acquisition. *Mol. Cell. Proteomics MCP*, **5**, 144–156.
